# In Silico Mining of Terpenes from Red-Sea Invertebrates for SARS-CoV-2 Main Protease (M^pro^) Inhibitors

**DOI:** 10.3390/molecules26072082

**Published:** 2021-04-05

**Authors:** Mahmoud A. A. Ibrahim, Alaa H. M. Abdelrahman, Tarik A. Mohamed, Mohamed A. M. Atia, Montaser A. M. Al-Hammady, Khlood A. A. Abdeljawaad, Eman M. Elkady, Mahmoud F. Moustafa, Faris Alrumaihi, Khaled S. Allemailem, Hesham R. El-Seedi, Paul W. Paré, Thomas Efferth, Mohamed-Elamir F. Hegazy

**Affiliations:** 1 Computational Chemistry Laboratory, Chemistry Department, Faculty of Science, Minia University, Minia 61519, Egypt; a.abdelrahman@compchem.net (A.H.M.A.); kh.abdeljawaad@compchem.net (K.A.A.A.); 2 Chemistry of Medicinal Plants Department, National Research Centre, 33 El-Bohouth St., Dokki, Giza 12622, Egypt; tarik.nrc83@yahoo.com; 3 Molecular Genetics and Genome Mapping Laboratory, Genome Mapping Department, Agricultural Genetic Engineering Research Institute (AGERI), Agricultural Research Center (ARC), Giza 12619, Egypt; matia@ageri.sci.eg; 4 National Institute of Oceanography & Fisheries, NIOF, Cairo 11516, Egypt; coralreef_noif1@yahoo.com (M.A.M.A.-H.); emelkady@yahoo.com (E.M.E.); 5 Department of Biology, College of Science, King Khalid University, Abha 9004, Saudi Arabia; hamdony@yahoo.com; 6 Department of Botany & Microbiology, Faculty of Science, South Valley University, Qena 83523, Egypt; 7 Department of Medical Laboratories, College of Applied Medical Sciences, Qassim University, Buraydah 51452, Saudi Arabia; f_alrumaihi@qu.edu.sa (F.A.); k.allemailem@qu.edu.sa (K.S.A.); 8 Department of Molecular Biosciences, The Wenner-Gren Institute, Stockholm University, S-106 91 Stockholm, Sweden; 9 Department of Chemistry, Faculty of Science, El-Menoufia University, Shebin El-Kom 32512, Egypt; 10 International Research Center for Food Nutrition and Safety, Jiangsu University, Zhenjiang 212013, China; 11 Department of Chemistry & Biochemistry, Texas Tech University, Lubbock, TX 79409, USA; paul.pare@ttu.edu; 12 Department of Pharmaceutical Biology, Institute of Pharmaceutical and Biomedical Sciences, Johannes Gutenberg University, Staudinger Weg 5, 55128 Mainz, Germany; efferth@uni-mainz.de

**Keywords:** drug discovery, marine natural products, molecular docking, molecular dynamics, SARS-CoV-2 main protease, virtual drug screening

## Abstract

Severe acute respiratory syndrome coronavirus 2 (SARS-CoV-2) is the causative agent for the COVID-19 pandemic, which generated more than 1.82 million deaths in 2020 alone, in addition to 83.8 million infections. Currently, there is no antiviral medication to treat COVID-19. In the search for drug leads, marine-derived metabolites are reported here as prospective SARS-CoV-2 inhibitors. Two hundred and twenty-seven terpene natural products isolated from the biodiverse Red-Sea ecosystem were screened for inhibitor activity against the SARS-CoV-2 main protease (M^pro^) using molecular docking and molecular dynamics (MD) simulations combined with molecular mechanics/generalized Born surface area binding energy calculations. On the basis of *in silico* analyses, six terpenes demonstrated high potency as M^pro^ inhibitors with Δ*G*_binding_ ≤ −40.0 kcal/mol. The stability and binding affinity of the most potent metabolite, erylosides B, were compared to the human immunodeficiency virus protease inhibitor, lopinavir. Erylosides B showed greater binding affinity towards SARS-CoV-2 M^pro^ than lopinavir over 100 ns with Δ*G*_binding_ values of −51.9 vs. −33.6 kcal/mol, respectively. Protein–protein interactions indicate that erylosides B biochemical signaling shares gene components that mediate severe acute respiratory syndrome diseases, including the cytokine- and immune-signaling components *BCL2L1*, *IL2*, and *PRKC*. Pathway enrichment analysis and Boolean network modeling were performed towards a deep dissection and mining of the erylosides B target–function interactions. The current study identifies erylosides B as a promising anti-COVID-19 drug lead that warrants further *in vitro* and *in vivo* testing.

## 1. Introduction

Severe acute respiratory syndrome coronavirus 2 (SARS-CoV-2) belongs to the family Coronaviridae (genus Betacoronavirus; subgenus Sarbecovirus) with a viral envelope encapsulating a single-stranded RNA genome. While most coronaviruses infect mammals and birds, the animal host responsible for the transmission of SARS-CoV-2 to men has yet to be identified [1,2,3].

The SARS-CoV-2 genome contains 11 genes encoding 29 proteins and peptides (www.ncbi.nlm.nih.gov/nuccore/NC_045512.2?report=graph). Four proteins constitute the viral structure, including the spike or S protein responsible for binding with the host cell receptor [4]. The S protein binds to the angiotensin-converting enzyme 2 (ACE2), which is a necessary step for viral entry into the cell. Two currently employed anti-SARS-CoV-2 vaccines utilize nanoparticle-encapsulated mRNA coding for the spike protein. These vaccines activate human immune responses to target the S protein, and about 95% of vaccinated people are protected from severe courses of the Coronavirus disease 2019 (COVID-19) [5]. In the case of more contagious variants such as a strain originating from Brazil (known as P.1), the genome has 17 unique mutations, including three in the receptor-binding domain of the spike protein. Mutations in the S protein are thought to be responsible for the variable ability of the spike protein to bind to ACE2, and specifically, the P.1 mutant reveals increased affinity and penetration into the host. Recently, an increasing number of escape mutations in the spike protein threaten the effectiveness of vaccines [6].

In seeking alternative approaches to vaccination, other SARS-CoV-2 proteins can be investigated that control the virus replication. Essential nonstructural proteins are initially expressed as two large polyproteins that are then processed into 16 peptide components. The main protease (M^pro^) initially cleaves the polyproteins into 11 fragments. Inhibitors that block the catalytic M^pro^ activity effectively interrupt the viral replication. One such inhibitor has been recently identified [7]. The crystal structure of SARS-CoV-2 main protease provides a basis for the design of improved alpha-ketoamide inhibitors [7] and M^pro^ appears to be a promising target for designing novel small molecule inhibitors. Since that original study, several promising inhibitor leads have been identified using *in silico* enzyme modeling [8]. Structure-based computational modeling of ligand–receptor interactions was used by Ibrahim et al. to identify potential M^pro^ inhibitors [9,10,11,12,13]. Natural products hold a vital role in discovering novel and effective therapeutics to combat the present COVID-19 pandemic. Among natural products, flavonoids, alkaloids, and terpenoids have attracted great attention as prospective SARS-CoV-2 inhibitors [14,15,16]. Recognizing that marine invertebrates are promising organisms for biologically active metabolites including anti-inflammatory, antibacterial, antifungal, antimalarial, antitumor, and antiviral activity [17,18], here biologically active terpene metabolites identified from a coral reef community unique to the Red Sea [19] were screened for *in silico* binding affinities against SARS-CoV-2 M^pro^. Previously characterized metabolites from this natural-product pool include alismol and aromadendrane sesquiterpenes derived from *Litophyton arboretum* [20] that exhibit inhibitory activity against the HIV-1 protease (HIV-1 PR) (IC_50_ 7 μM); palustrol, a sesquiterpene from *Sarcophyton trocheliophorum* that has antibacterial activity (MIC 6.6–11.1 μM) [21]; and 12(S)-Hydroperoxylsarcoph-10-ene, a cembrane diterpene from *Sarcophyton glaucum* that was reported to exhibit potent anticancer activity via the inhibition of Cyp1A activity (*p* < 0.01) with IC_50_ values of 2.7 nM [22]. On the basis of the predicted docking scores, the most potent inhibitors are submitted to molecular dynamics (MD) simulations combined with binding energy calculations using a molecular mechanics/generalized Born surface area approach.

## 2. Results and Discussion

Since the main protease (M^pro^) of SARS-CoV-2 plays an indispensable role in viral reproduction, small molecules were screened based on *in silico* molecular docking calculations and MD simulations for prospective M^pro^ inhibitors. Marine natural products identified from the Red Sea provided the source for metabolite screening.

### 2.1. Molecular Docking

**Figure 1:**
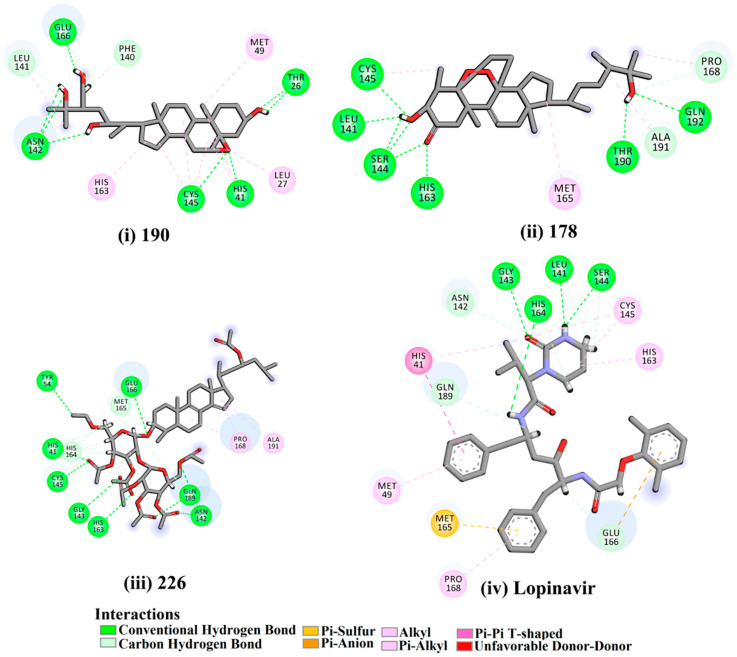
2D representations of the predicted binding modes of MNPs (**i**) 190, (**ii**) 178, (**iii**) 226, and (**iv**) lopinavir towards SARS-CoV-2 main protease (M^pro^).

Two hundred and twenty-seven terpene natural products isolated from the biodiverse Red-Sea ecosystem were screened against the SARS-CoV-2 main protease (M^pro^) using molecular docking technique. Molecular docking calculations resulted in 27 of the screened compounds exhibiting a higher binding affinity than lopinavir: an inhibitor of SARS-CoV-2 main protease (M^pro^) that was proposed as a treatment for COVID-19 on the basis of *in vitro* activity, preclinical studies, and observational studies [23]. While docking scores ranged from −4.3 to −12.3 kcal/mol, 12% of the compounds scored below −9.8 kcal/mol (Table S1). AutoDock4.2.6 software was utilized to carry out all molecular docking calculations. Binding affinities, 2D chemical structures, and features of the 27 most promising natural products towards SARS-CoV-2 M^pro^ are summarized in Table 1. 2D docking positions with proximal amino acid residues within the M^pro^ active site are depicted in Figure S1. Most of these compounds demonstrate similar M^pro^ binding modes within the binding pocket, forming hydrogen bonds with CYS145, HIS164, and GLU166, which can account for the high binding affinities (Table 1 and Figure S1). The 2D and 3D representations of the interactions of the top three potent marine natural products (MNPs) and lopinavir with key amino acid residues of SARS-CoV-2 M^pro^ are depicted in Figure 1 and Figure S2, respectively.

Depresosterol (190) isolated from *Lobophytum depressum*, exhibited the highest binding affinity of the compounds screened against SARS-CoV-2 M^pro^, with a docking score of −12.3 kcal/mol. *in silico* M^pro^ binding in the active site indicated that the methanolic hydroxyl group exhibited two hydrogen bonds with a backbone carboxylate of GLU166 with bond lengths of 1.99 and 2.55 Å, respectively (Figure 1 and Table 1). In addition, the hydroxyl unit of 2-methylpropan-2-ol affords three hydrogen bonds with a backbone NH and carbonyl group of ASN142 with bond lengths of 2.24, 2.68, and 2.04 Å, respectively (Figure 1 and Table 1). Moreover, the hydroxy group of 2-propanol exhibited a hydrogen bond with the backbone carbonyl group of ASN142 with a bond length of 1.96 Å (Figure 1, Figure S2 and Table 1). The oxygen of the oxirane ring interacted with the backbone imidazole ring of HIS41, and the thiol group of CYS145 with bond lengths of 2.17 and 2.70 Å, respectively (Figure 1 and Table 1). The hydroxy group of the cyclohexanol ring contributed two hydrogen bonds with NH and the carbonyl group of TYR26 with bond lengths of 2.15 and 2.66 Å, respectively (Figure 1 and Table 1).

3β-25-Dihydroxy-4-methyl-5α,8α-epidioxy-2-ketoergost-9-ene (178) isolated from *Sinularia candidula*, exhibited the second highest binding affinity of the compounds screened against SARS-CoV-2 M^pro^ with a docking score of −12.2 kcal/mol. *in silico* M^pro^ binding in the active site indicated that the hydroxy group of the hydroxycyclohexanone ring participates in four hydrogen bonds with the backbone carbonyl of LEU141, OH and NH of SER144, and NH of CYS145 with bond lengths of 2.08, 1.97, 2.28, and 2.49 Å, respectively (Figure 1 and Table 1). Moreover, the carbonyl group of the hydroxycyclohexanone ring demonstrates two hydrogen bonds with an imidazole ring of HIS163 and backbone OH of SER144 with bond lengths of 2.08 and 2.66 Å, respectively (Figure 1 and Table 1). The hydroxy group of 2-methylpropan-2-ol displays two hydrogen bonds with a backbone carbonyl group of THR190 and NH of GLN192 with bond lengths of 1.80 and 2.26 Å, respectively (Figure 1 and Table 1).

Erylosides B (226) isolated from *Erylus lendenfeldi* also exhibited a high binding affinity against SARS-CoV-2 M^pro^ with a docking score of −12.1 kcal/mol. *in silico* M^pro^ binding in the active site indicated that the five carbonyl groups of methyl acetates exhibit seven hydrogen bonds with a backbone OH of TYR54, imidazole ring of HIS41, SH of CYS145, imidazole ring of HIS163, NH of ASN142, NH of GLY143, and NH_2_ of GLN189 with bond lengths of 3.05, 1.96, 2.04, 1.93, 1.75, 2.82, and 1.91 Å, respectively (Figure 1 and Table 1). Moreover, the oxygen of the (cyclohexyloxy) cyclohexane ring forms a hydrogen bond with the backbone NH of GLU166 with a bond length of 2.37 Å (Figure 1 and Table 1). The oxygen atom of the methoxy group is also hydrogen bound to the NH of ASN142 with a bond length of 2.96 Å (Figure 1 and Table 1). Compared with 190, 178, and 226, lopinavir presented a similar binding affinity towards M^pro^ with a docking score of −9.8 kcal/mol. Scrutinizing the binding mode of lopinavir inside the active site of M^pro^ revealed that the NH of the tetrahydro-1-methylpyrimidin-2(1H)-one ring exhibited two hydrogen bonds with a backbone hydroxy group of SER144 and a carbonyl group of LEU141 with bond lengths of 3.09 and 1.96 Å, respectively.

The carbonyl group of the tetrahydro-1-methylpyrimidin-2(1H)-one ring was also observed to form a hydrogen bond with the backbone NH of GLY143 with a bond length of 2.01 Å, and the NH of the methylacetamide group participates in a hydrogen bond with the carbonyl group of HIS164 with a bond length of 2.62 Å (Figure 1 and Table 1). The current results provide quantitative data of the binding affinities of 190, 178, 226, and lopinavir as promising SARS-CoV-2 M^pro^ inhibitors.

### 2.2. Molecular Dynamics (MD) Simulations

**Figure 2:**
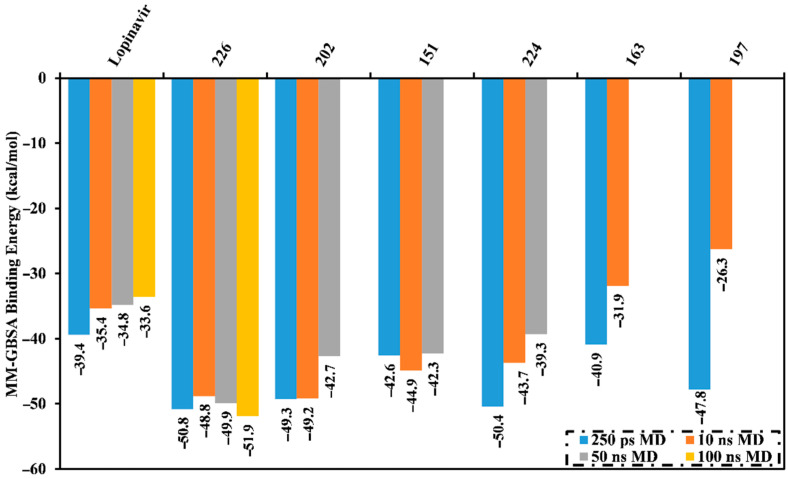
Average molecular mechanics/generalized Born surface area (MM/GBSA) binding energies for lopinavir and the top natural products complexed with M^pro^ over 250 ns in an implicit water solvent, and 10 ns, 50 ns, and 100 ns molecular dynamics (MD) simulations in an explicit water solvent.

MD simulations probe the stability of the ligand–enzyme complexes, conformational flexibilities, structural details, and the dependability of ligand–enzyme affinities [24,25]. Therefore, those natural products with low M^pro^ docking scores were submitted for MD simulations followed by binding energy calculations. The simulations were conducted with an implicit water solvent for 250 ps, and the molecular mechanics/generalized Born surface area (MM/GBSA) approach was applied to estimate the corresponding binding affinities; thereby diminishing the time and computational costs (see computational methodology section for details). The calculated MM/GBSA binding energies for the pre-screened natural products are summarized in Table S2. Six compounds had lower binding energies (Δ*G*_binding_) than lopinavir (calc. −39.4 kcal/mol). These metabolites were further subjected to a 10 ns MD simulation in an explicit water solvent to obtain more accurate M^pro^ binding affinities. MD/MM/GBSA binding energies were estimated (Figure 2). Four of these compounds exhibited lower binding energies (Δ*G*_binding_) compared to lopinavir (calc. −35.4 kcal/mol). Moreover, those potent MNPs were chosen and submitted for 50 ns MD simulations in the explicit water solvent, and the corresponding binding energies were evaluated (Figure 2). Only erylosides B (226) exhibited steady binding throughout the simulation, while 202, 151, and 224 showed an increase in MM/GBSA binding energies over the simulated time. For instance, the calculated MM/GBSA binding energies for 226 against M^pro^ were −50.8, −48.8 and −50.0 kcal/mol over 250 ps implicit-solvent MD, 10 ns explicit-solvent MD, and 50 ns explicit-solvent MD simulations, respectively. This shows the importance of long MD simulations to predict metabolite-M^pro^ binding affinities. Therefore, MD simulations for the 226-M^pro^ complex were prolonged to 100 ns, and the corresponding MM/GBSA binding energy was calculated (Figure 2).

**Figure 3:**
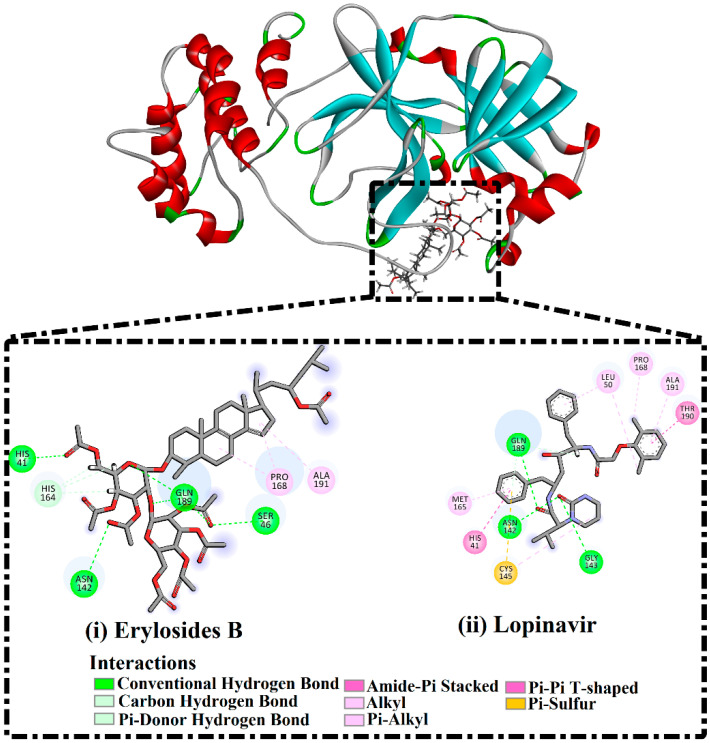
2D representations of binding modes of (**i**) erylosides B (226)- and (**ii**) lopinavir-M^pro^ complexes according to an average structure over a 100 ns MD simulation.

No appreciable variance between the calculated MM/GBSA binding energy for the 226-M^pro^ complex throughout the 50 ns MD simulation and the corresponding MM/GBSA binding energy throughout the 100 ns MD simulation were observed (Figure 2). Compared with lopinavir, 226 showed a higher binding affinity towards M^pro^ over the 100 ns MD simulation with an average Δ*G*_binding_ of −51.9 kcal/mol. Hydrogen bonding, pi-based, hydrophobic, and van der Waals interactions with essential amino acid residues within the M^pro^ active site are all thought to contribute to the strong binding constant. The 2D and 3D representations for 226 and lopinavir within the M^pro^ active site over 100 ns are shown in Figure 3 and S3, respectively. Interestingly, 226 maintained hydrogen bonding with the proximal amino acid residues of M^pro^ over the 100 ns MD course (Figure 3). Structural insights into the binding mode of the 226 with the M^pro^ demonstrated that the carbonyl groups of methyl acetates form four hydrogen bonds with the backbone imidazole of the HIS41, OH of SER146, the carbonyl group of ASN142, and NH2 of GLN189, with bond lengths of 2.72, 2.76, 3.37, and 1.89 Å, respectively (Figure 3); while the oxygen of the (cyclohexyloxy) cyclohexane ring forms hydrogen bonds with the backbone NH2 of GLN189 with a bond length of 2.62 Å (Figure 3). Furthermore, the oxygen of tetrahydro-2H-pyran bonds with the backbone NH2 of GLN189 with a bond length of 2.06 Å (Figure 3).

While lopinavir also displayed high M^pro^ binding energy over the 100 ns MD simulation at Δ*G*_binding_ −33.6 kcal/mol, only three hydrogen bonds were observed with the proximal amino acid residues of M^pro^ (Figure 3). A comparison of the erylosides B (226) and lopinavir revealed that the binding affinity of erylosides B (226) was approximately two times higher than that of lopinavir.

The estimated MM/GBSA binding energies were further decomposed into separate components to indicate the force in the binding of M^pro^ with erylosides B (226) and lopinavir (Table 2). *E*_vdw_ was a significant contributor to the erylosides B (226)- and lopinavir-M^pro^ binding affinities with average values of −71.2 and −45.6 kcal/mol, respectively. *E*_ele_ was efficient with an average value of −30.5 and −22.1 kcal/mol for the erylosides B (226)- and lopinavir-M^pro^ binding affinities, respectively.

**Figure 4:**
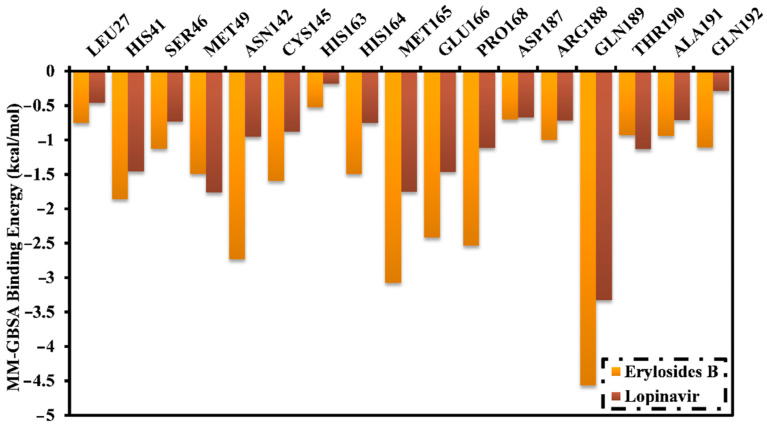
Energy contributions (kcal/mol) for M^pro^ amino acid residues to the binding free energy of erylosides B (226) and lopinavir.

The binding energies of erylosides B (226)- and lopinavir-M^pro^ complexes were further decomposed at the per-residue level and the residues with absolute energy contribution < −0.50 kcal/mol were shown (Figure 4). MET165, GLU166, PRO168, and GLN189 in the M^pro^ complex favorably participate with erylosides B (226) and lopinavir. There was a considerable contribution by GLN189 to the total binding free energy with values of −4.6 and −3.3 kcal/mol for erylosides B (226) and lopinavir, respectively. Additionally, the hydrophobic residues share greater energies as a result of the formation of the hydrophobic interactions between erylosides B (226) and hydrophobic residues.

### 2.3. Post-Dynamics Analyses

To further confirmed the stability and behavior of erylosides B (226) complexed with M^pro^, structural and energetic analyses were executed during 100 ns MD simulations and compared to those of lopinavir. Control of the structural stability of the studied system was accomplished by investigating root-mean-square deviation (RMSD), center-of-mass (CoM) distance, hydrogen bond length, and binding energy per frame.

#### 2.3.1. Binding Energy per Frame

**Figure 5:**
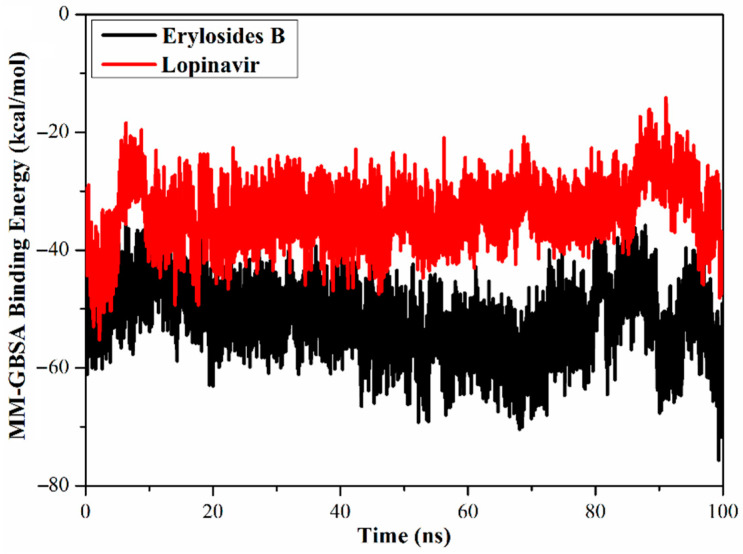
Calculated MM/GBSA binding energy per frame for erylosides B (black) and lopinavir (red) with M^pro^ over 100 ns MD simulations.

The global structural stability of erylosides B (226) and lopinavir in complex with M^pro^ was estimated over the 100 ns MD simulations by measuring the correlation between the binding energy per frame and time (Figure 5). Overall stabilities for erylosides B (226) and lopinavir exhibited average binding energies (Δ*G*_binding_) of −51.9 and −33.6 kcal/mol, respectively. According to this analysis, all complexes conserved stability throughout the 100 ns MD simulation.

#### 2.3.2. Hydrogen Bond Length

Hydrogen bond analyses for erylosides B (226)- and lopinavir-M^pro^ complexes over a 100 ns MD simulation were performed (Table 3). Four hydrogen bonds with GLN189, GLU166, CYS145, and ASN142 were observed within the erylosides B (226)-M^pro^ complex. Average bond lengths were 2.9, 2.8, 2.9, and 2.7 Å, with occupation percentages of 96, 92, 91, and 83%, respectively. In contrast, two hydrogen bonds were detected for the lopinavir-M^pro^ complex, with GLN189 and GLY143 showing an average bond length of 2.8 and 2.7 Å and an occupation percentage of 85.6 and 75.6%, respectively. Overall, these hydrogen bond analyses supported the finding of high stability for the erylosides B (**226**)-M^pro^ complex compared to lopinavir-M^pro^.

#### 2.3.3. Center-of-Mass Distance

**Figure 6:**
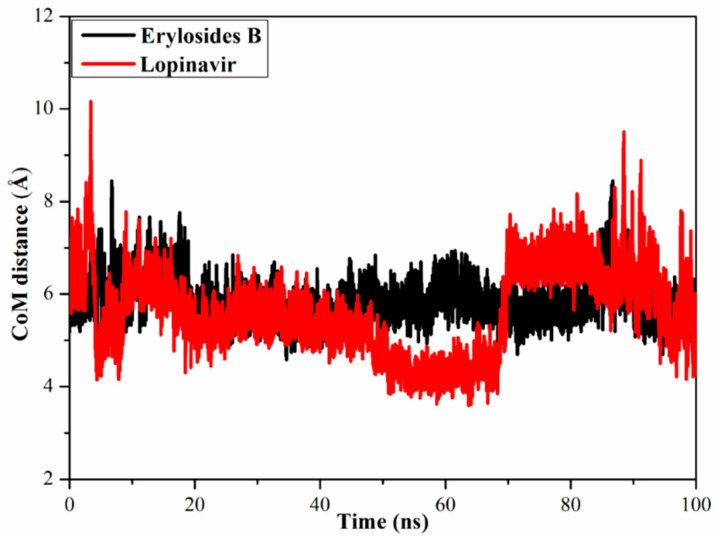
Center-of-mass (CoM) distances (in Å) between erylosides B (black) and lopinavir (red) and GLN189 of M^pro^ over a 100 ns MD simulation.

To gain a more in-depth insight into the stability of ligand-M^pro^ during the MD simulation, center-of-mass (CoM) distances were measured (Figure 6). Interestingly, CoM distances were consistent for the erylosides B (226)-M^pro^ complex compared to lopinavir-M^pro^, with average values of 5.6 vs. 5.9 Å, respectively. This suggests that erylosides B (226) bound more tightly to the M^pro^ complex than lopinavir.

#### 2.3.4. Root-Mean-Square Deviation

**Figure 7:**
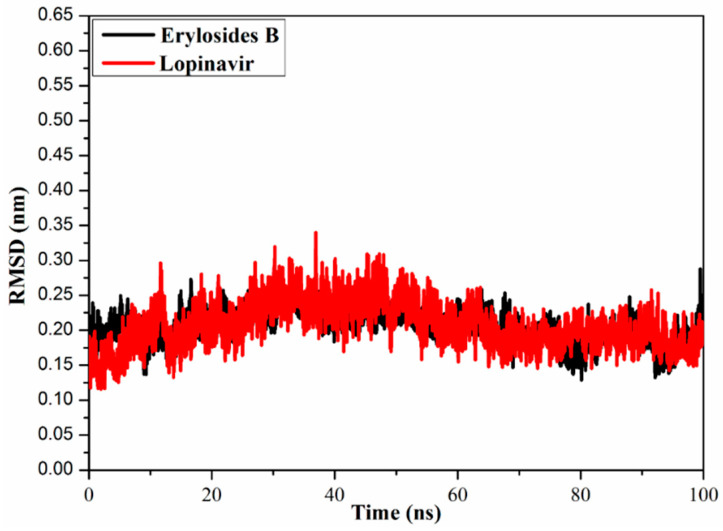
Root-mean-square deviation (RMSD) of the backbone atoms from the initial structure of erylosides B (black) and lopinavir (red) with M^pro^ throughout a 100 ns MD simulation.

To watch the structural stability of the erylosides B (226)- and lopinavir-M^pro^ complexes, the root-mean-square deviation (RMSD) values of the backbone atoms of the whole complex were estimated (Figure 7). Unambiguously, the estimated RMSD values for the investigated complexes remained below 0.20 nm over the 100 ns MD simulations. The erylosides B (226)- and lopinavir-M^pro^ complexes reached the steady state in the first 10 ns MDs, and continued in an almost stationary state until the end of the simulations. The current results emphasized that the erylosides B (226) is tightly bonded and does not impact the overall topology of SARS-CoV-2 M^pro^.

### 2.4. Molecular Target Prediction and Network Analysis

**Figure 8:**
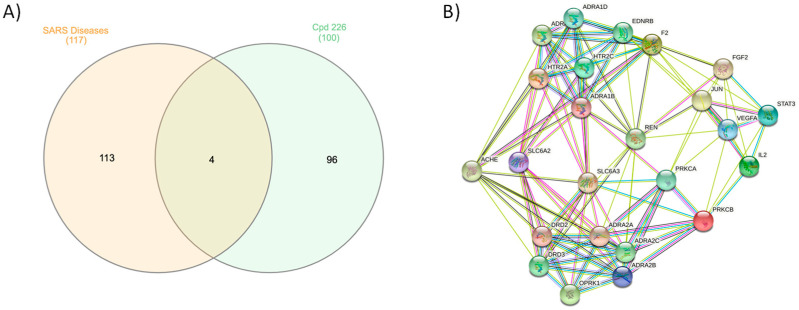
(**A**) Venn diagram analysis for erylosides B (226) with SARS disease genes and (**B**) STRING PPI network for the top targets identified by network analyzer for erylosides B (226) as a M^pro^ inhibitor.

Erylosides B (226) protein targets associated with severe acute respiratory syndrome diseases were predicted using a SwissTargetPrediction DisGeNET online tool. One hundred and seventeen genes were identified using Venn diagram comparison analysis. Commonly shared genes for erylosides B (226) were *BCL2L1, IL2, PRKCA*, and *PRKCB* (Figure 8). Several studies reported that these four genes alter cytokine levels and immune functions in patients with COVID-19 infections and related conditions [26,27]. Erylosides B (226) predicted gene targets were also analyzed via a STRING protein–protein interaction (PPI) network and visualized by Cytoscape 3.8.0. The top 20 scored genes for erylosides B (226) also included *BCL2L1, IL2, PRKCA*, and *PRKCB* (Table S3).

### 2.5. Pathway Enrichment Analysis (PEA)

**Figure 9:**
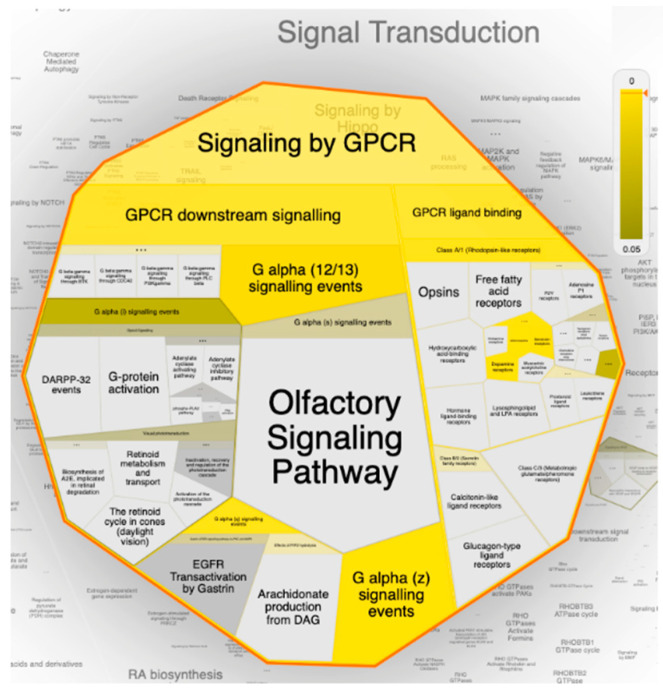
The Voronoi treemap of the top pathway (signal transduction) influenced by the top 20 gene targets in response to erylosides B (226) in term of SARS-CoV-2 infection. The color highlights the over-representation of that pathway in the input dataset. Light grey signifies pathways that are not significantly over-represented.

Toward a deep dissection and mining of the erylosides B (226) target–function interactions, PEA analysis and Boolean network modeling were conducted. A Voronoi treemap of erylosides B (226) top targeted pathway affected by the top 20 gene targets in response to erylosides B (226) in terms of SARS-CoV-2 infection was constructed (Figure 9). Moreover, a Reactome hierarchy map was built to provide a genome-wide representation of the pathways influenced in response to erylosides B treatment (Figure S4). Remarkably, among the top 20 enriched pathways from the Reactome–PEA analyses and signal transduction, immune system and homeostasis signaling pathways were found to be the major pathways targeted by erylosides B (226), with a high significance (FDR < 0.00001%) (Table S4).

Interestingly, under signal transduction, it was found that the G-protein coupled receptor (GPCR) signaling pathway was the most enriched pathway influenced by erylosides B (226) treatment within the human genome. Mining of the Reactome–PEA analysis results emphasized that a set of 15 genes (*OPRK1, HTR2C, PRKCA, HTR2A, ADRA1D, PRKCB, DRD2, DRD3, ADRA2A, EDNRB, ADRA1A, ADRA2B, ADRA1B, ADRA2C*, and *F2*) were significantly modulated as biological targets to erylosides B (226) as potent SARS-CoV-2 inhibitor. Additionally, the Reactome–PEA results revealed that these genes were found to interact with other genes/interactors, including *P16220, P52292, P00533, P12931, P07550, P35414, P01019, P30559, P14416,* and *O15354*.

**Figure 10:**
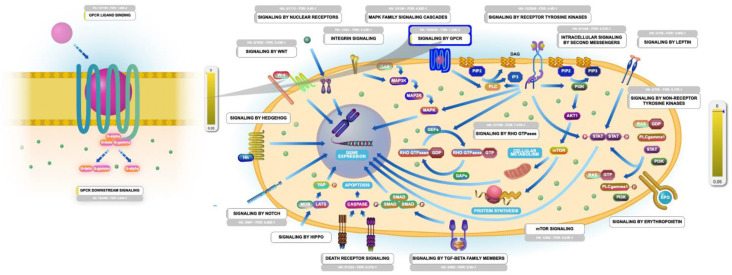
Graphic representation of the Reactome pathways influenced as a response to erylosides B (226) in term of SARS-CoV-2 infection. The representation showing the G-protein coupled receptor (GPCR) signaling pathway as the most enriched pathway influenced by erylosides B (226) treatment in the human genome.

GPCRs are a massive family of surface transmembrane receptors in a human cell capable of responding to numerous stimulants and promoting a cascade of cellular functions. According to the GPCRs’ pharmacological attributes, they are divided into four prominent families: the rhodopsin-like receptors family, the secretin receptors family, the metabotropic glutamate/pheromone receptors family, and the frizzled receptors family [28]. Remarkably, the Reactome–PEA analyses results revealed that almost all GPCR prominent families were significantly influenced by erylosides B (Figure 10). Many recent studies found that SARS-CoV-2 may compromise the GPCR signaling pathway and contribute to pulmonary edema’s pathophysiology [29]. Additionally, recent reports speculated that SARS-CoV-2 might hijack the GPCR signaling pathway— mimicking a previous case report in cholera toxin, ADP-ribosylation Gαs—to activate CFTR and switch on Cl^-^ secretion, eventually leading to dysregulation of lung ion and fluids transmission, which ultimately led to lung edema in COVID-19 patients [30].

## 3. Materials and Methods

### 3.1. M^pro^ Preparation

The resolved three-dimensional (3D) structure of SARS-CoV-2 main protease (M^pro^) with a resolution of 2.16 Å (PDB code: 6LU7 [31]) complexed with peptidomimetic inhibitor (N3) was downloaded and used as a template for all molecular docking and MD calculations. The protein structure was prepared by removing all heteroatoms, crystallographic waters, and ions, conserving only the amino acid residues. The protonation states of amino acid residues of M^pro^ were assigned using H++ web server, and all missing hydrogen atoms were inserted [32]. The pKa values of M^pro^ amino acid residues were estimated under physical conditions of salinity = 0.15, internal dielectric constant = 10, pH = 7, and external dielectric constant = 80.

### 3.2. Inhibitor Preparation

The chemical structures of the 227 scrutinized marine natural products (MNPs) described in literature as Red-Sea terpenes were obtained in SDF format from the PubChem database (https://pubchem.ncbi.nlm.nih.gov). All MNPs were processed using Omega2 software to generate the three-dimensional (3D) structures of the studied molecules [33,34]. Merck Molecular Force Field 94 (MMFF94S), implemented inside SZYBKI software [35,36], was applied to minimize the geometry of the investigated MNPs. The two-dimensional (2D) chemical structures of the scrutinized molecules are presented in Table S1.

### 3.3. Molecular Docking

All molecular docking calculations were executed using AutoDock4.2.6 software [37]. The pdbqt file of SARS-CoV-2 main protease (M^pro^) was prepared on the basis of the AutoDock protocol [38]. The maximum number of energy evaluations (*eval*) and the genetic-algorithm number (*GA*) were adjusted to 25,000,000 and 250, respectively. All other docking parameters were conserved at default. The grid box dimensions were adjusted to 60 Å × 60 Å × 60 Å to cover the SARS-CoV-2 M^pro^ binding pocket. Moreover, the grid spacing value was set to 0.375 Å. The coordinates of the grid center were located at −13.069, 9.740, and 68.490 in the x, y, and z directions, respectively. The atomic partial charges of the investigated MNPs were calculated using the Gasteiger method [39]. The predicted binding poses for each investigated MNP were processed by the built-in clustering analysis (1.0 Å RMSD tolerance), and the conformation with the lowest energy within the most massive cluster was picked out as a representative pose.

### 3.4. Molecular Dynamics Simulations

AMBER16 software was employed to perform all MD simulations for the most potent MNPs complexed with SARS-CoV-2 M^pro^ [40]. M^pro^ and the investigated MNPs were determined using AMBER force field 14SB [41] and General AMBER force field (GAFF2) [42], respectively. In the present study, implicit-solvent and explicit-solvent MD simulations were performed. In the implicit-solvent MD simulations, the atomic partial charges of the investigated MNPs were evaluated using the AM1-BCC method [43]. No periodic boundary conditions and no cut-off were applied for nonbonded interactions (specifically, a cutoff value of 999 Å was employed). Furthermore, the solvent influence was estimated using igb = 1 solvent model [44]. Energy minimization was first carried out on the docked MNP-M^pro^ complexes for 500 steps, and the minimized complexes were then gently heated from 0 K to 300 K over 10 ps under NVT condition using a Langevin thermostat. Finally, the production stage was executed over 250 ps, and snapshots were assembled every 1 ps, giving 250 snapshots. In the present study, all implicit-solvent MD simulations were conducted using the CPU version of pmemd (pmemd.MPI) implemented inside AMBER16.

In explicit-solvent MD simulations, the atomic partial charges of the investigated MNPs were estimated using the restrained electrostatic potential (RESP) approach at the HF/6-31G* level with the help of Gaussian09 software [45,46]. The TIP3P water model with periodic boundary conditions was utilized to solvate the MNP-M^pro^ complexes in a cubic water box with a minimum distance to the box edge of 15 Å. Energy minimizations for 5000 steps on the solvated MNP-M^pro^ complexes were performed using combined steepest and conjugate gradient methods. The minimized complexes were then smoothly heated from 0 to 300 K over 50 ps and a restraint of 10 kcal mol^−1^ Å^−1^ was applied on the main protease. The complexes were then equilibrated under NPT conditions for 1 ns. The production stage was then performed for each investigated M^pro^-inhibitor complex over simulation times of 10 ns, 50 ns, and 100 ns. The particle mesh Ewald (PME) method was applied to estimate the long-range electrostatic forces and energies. Cut-off for coulombic interactions with a value of 12 Å was considered [47]. To maintain the temperature at 298 K, Langevin dynamics with a gamma_ln collision frequency set to 1.0 was utilized. A Berendsen barostat with a pressure relaxation time of 2 ps was applied for the pressure control [48]. To constrain all bonds including hydrogen atoms, a SHAKE algorithm with a time step of 2 fs was utilized [49]. For binding energy calculations and post-dynamics analyses, energy values and coordinates were collected every 10 ps over the production stage. All explicit-solvent MD simulations were executed utilizing the GPU version of pmemd (pmemd.cuda) implemented inside AMBER16. All *in silico* calculations involving quantum mechanics, molecular docking calculations, and MD simulations were carried out on the CompChem GPU/CPU cluster (hpc.compchem.net). BIOVIA DS Visualize 2020 was utilized to generate all molecular graphics [50].

### 3.5. Free Binding Energy Calculations

The molecular mechanics/generalized Born surface area (MM/GBSA) approach [51] was applied to evaluate the binding free energies of the most potent MNPs complexed with SARS-CoV-2 M^pro^. To appoint the polar solvation energy, the modified GB model proposed by Onufriev [52] (igb = 2) was employed. The uncorrelated snapshots collected throughout the MD simulations were subjected to MM/GBSA (Δ*G*_binding_) energy calculations, which estimated as follows:

ΔG_binding_ = G_Complex_ − (G_MNP_ + G_Mpro_) (1)

where the energy term (*G*) is evaluated as:

*G* = *E*_ele_ + *E*_vdw_ + *G*_SA_ + *G*_GB_ (2)

*E*_ele_ and *E*_vdw_ and are electrostatic and van der Waals energies, respectively. *G*_SA_ is the nonpolar contribution to the solvation-free energy from the solvent-accessible surface area (SASA). *G*_GB_ is the electrostatic solvation free energy calculated from the generalized Born equation. A single-trajectory approach was applied, in which the coordinates of each MNP-M^pro^, M^pro^, and MNP were acquired from a single trajectory. Because of the expensive computational request and low prediction thoroughness in most cases, entropy calculations were ignored [53,54].

### 3.6. Protein–Protein Interaction and Pathway Enrichment Analysis (PEA)

According to the structural resemblance of recognized inhibitor–target integrations, target forecasting of the most auspicious marine natural products was carried out utilizing the online web-based tools of SwissTargetPredicition (http://www.swisstargetprediction.ch). Moreover, the DisGeNET online database (https://www.disgenet.org) was applied to gather the accessible database for Severe Acute Respiratory Syndrome (SARS) diseases. The InteractiVenn online tool was employed to design Venn diagram [55]. A functional database of STRING for top predicted targets was applied to generate protein–protein interaction (PPI) network [56]. Cytoscape 3.8.2 was applied to examine target-function relation according to the network topological [57]. Furthermore, to explore all probable target–function relations for the top 20 SARS disease-related genes based on their network profiles, pathway enrichment analysis was achieved using Cytoscape 3.8.2 [57]. Lastly, the ReactomeFIViz tool was integrated for modeling and visualizing all potential drug–target interactions [58].

## 4. Conclusions

In the context of the COVID-19 pandemic, it is apparent that zoonotic diseases pose a huge risk to public health and economic stability. A lack of targeted treatments has spurred the exploration of novel drug leads for which computational approaches provide a relatively quick and cost-effective approach. Herein, marine natural products isolated from the Red Sea were screened *in silico* as potential M^pro^ inhibitors. On the basis of molecular docking calculations, MD simulations, and molecular mechanics/generalized born surface area binding energy calculations, erylosides B (226) demonstrated a favorable binding affinity with Δ*G*_binding_ < −51.0 kcal/mol with M^pro^. The stability of 226 complexed with M^pro^ was confirmed using energetic and structural analyses over the 100 ns MD simulation. Ultimately, we hypothesize that the utilization of erylosides B may represent a potential therapeutic approach against COVID-19 due to its useful capability to modulate the GPCR signaling pathway in COVID-19 patients. Experimental validation is expected to further identify the suitability of natural products such as erylosides B to serve as a SARS-CoV-2 inhibitor.

## Figures and Tables

**Table 1:** Estimated docking scores, 2D chemical structures, and binding features for lopinavir and the top 27 potent marine natural products (MNPs) towards SARS-CoV-2 main protease (M^pro^).

MNP Name	Plant Source	2D Chemical Structure	Docking Score (kcal/mol)	Binding Features ^a^
Lopinavir	--- ^b^	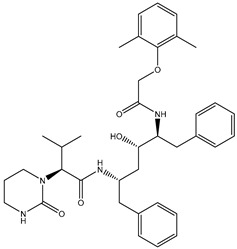	−9.8	HIS164 (2.62 Å), GLY143 (2.01 Å), LEU141 (1.96 Å), SER144 (3.09 Å)
Depresosterol (190)	*L. depressum*	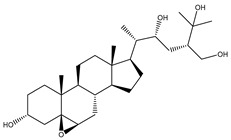	−12.3	THR26 (2.15, 2.66 Å), HIS41 (2.17 Å), CYS145 (2.70 Å), ASN142 (1.96, 2.04, 2.24 Å), GLU166 (1.99, 2.55 Å)
3β-25-Dihydroxy-4-methyl-5α,8α-epidioxy-2-ketoergost-9-ene (178)	*Sinularia candidula*	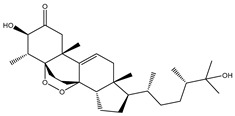	−12.2	LEU141 (2.08 Å), SER144 (1.97, 2.28, 2.66 Å), HIS163 (2.08 Å), CYS145 (2.49 Å), GLN192 (2.26 Å), THR190 (1.80 Å)
Erylosides B (226)	*E. lendenfeldi*	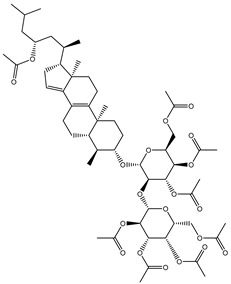	−12.1	HIS41 (1.96 Å), TYR54 (3.05 Å), AS142 (1.75, 2.96 Å), GLY143 (2.82 Å), CYS145 (2.04 Å), HIS163 (1.93 Å), GLU166 (2.37 Å), GLN189 (1.91 Å)
Sipholenol H (157)	*S. siphonella*	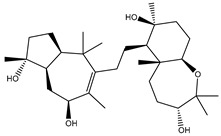	−12.0	GLY143 (1.74 Å), CYS145 (2.88 Å), HIS163 (2.56 Å), HIS164 (2.85 Å), MET165 (2.74 Å), THR190 (1.74, 2.17 Å), GLN192 (2.11 Å)
Dahabinone A (162)	*S. siphonella*	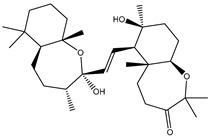	−11.9	CYS145 (2.34 Å), HIS41 (2.43 Å), GLU166 (2.01, 2.23, 2.38 Å), GLY143 (2.12 Å), GLN189 (2.03 Å)
Sipholenol I (174)	*S. siphonella*	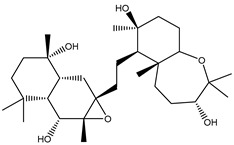	−11.8	GLY143 (2.04 Å), CYS145 (2.89 Å), HIS163 (2.89 Å), HIS164 (2.01 Å), GLU166 (1.62, 2.03, 2.52 Å)
Lobophytosterol (188)	*L. depressum*	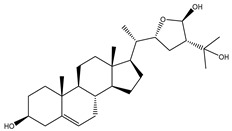	−11.5	TYR54 (2.24, 2.55 Å), ASN142 (1.91, 2.75 Å), GLU166 (1.86, 2.66 Å), ASP187 (2.94 Å)
(22R,24E,28E)-5β,6β-Epoxy-22,28-oxido-24-methyl-5αcholestan-3β,25,28-triol (191)	*L. depressum*	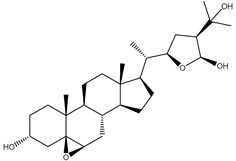	−11.4	THR26 (2.69 Å), HIS41 (2.14 Å), CYS145 (2.45 Å), ARG188 (2.03 Å), THR190 (2.06, 2.55 Å), GLN192 (2.26 Å)
Tasnemoxide A (144)	*D. erythraeanus*	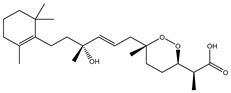	−11.4	CYS44 (2.10 Å), TYR54 (2.54, 2.97 Å), GLU166 (1.91, 1.97 Å)
Siphonellinol C (172)	*S. siphonella*	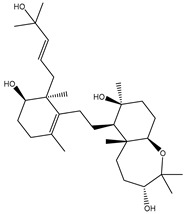	−11.3	GLY143 (1.95 Å), GLN189 (2.09 Å), THR190 (1.73, 2.41 Å), GLN192 (2.10 Å)
Siphonellinol-C-23-hydroperoxide (171)	*S. siphonella*	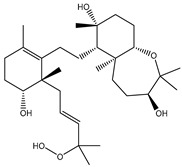	−11.2	GLY143 (2.00 Å), GLN189 (1.97 Å), THR190 (2.03, 2.43 Å), GLN192 (2.46 Å)
Erylosides K (224)	*Erylus lendenfeldi*	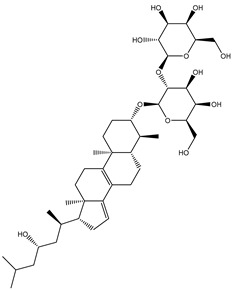	−11.1	GLU166 (3.03 Å), HIS163 (2.18 Å), HIS164 (2.26, 2.10 Å)
Sipholenol D (176)	*S. siphonella*	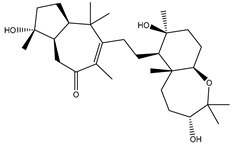	−11.0	THR190 (2.08 Å), HIS163 (2.48 Å), GLU166 (2.98 Å), GLN189 (1.91 Å)
Sipholenone A (175)	*S. siphonella*	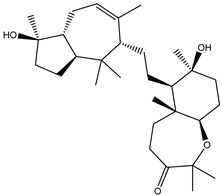	−11.0	GLY143 (1.93 Å), HIS163 (2.41 Å), HIS164 (2.54 Å), GLU166 (2.36 Å)
Neviotine B (158)	*S. siphonella*	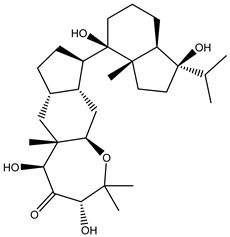	−10.9	ASN142 (2.26 Å), GLU166 (1.95 Å), GLN189 (1.80 Å), THR190 (2.57 Å)
Eryloside A (197)	*Genus Erylus*	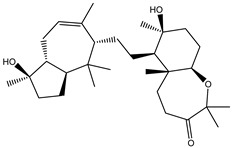	−10.7	ASN142 (2.32 Å), GLU166 (1.95 Å), THR190 (1.87 Å)
Sipholenone D (155)	*S. siphonella*	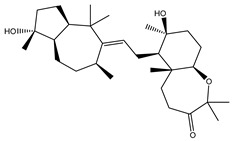	−10.7	GLN189 (1.77 Å), THR190 (1.83 Å), GLN192 (2.23 Å)
24-Methylcholestane-5-en-3β,25-diol (187)	*S. polydactyla*	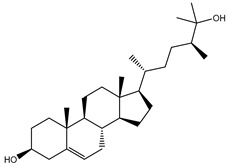	−10.6	MET49 (2.16 Å), PHE140 (1.87 Å), GLN189 (2.97 Å)
SipholenolA-4-O-3′,4′-dichlorobenzoate (151)	*S. siphonella*	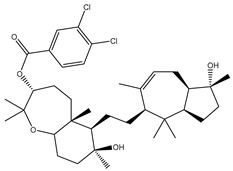	−10.5	HIS163 (2.30 Å), GLN189 (1.78 Å)
Stigmasterol (220)	*D. coccinea*	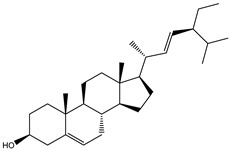	−10.5	MET49 (2.18 Å), GLN189 (2.97 Å)
Cholest-5-en-3β,7β-diol (206)	*A. dichotoma*	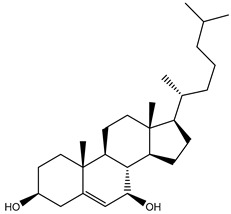	−10.3	MET49 (2.17 Å), GLN189 (1.94 Å)
Campesterol (221)	*D. coccinea*	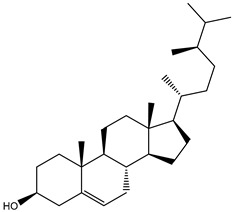	−10.3	MET49 (2.17 Å), GLN189 (3.01 Å)
Cholesterol (184)	*Dendronephthya*	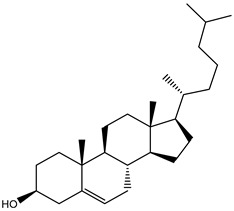	−10.3	MET49 (2.10 Å), GLN189 (2.97 Å)
Clionasterol (219)	*Dragmacidon coccinea*	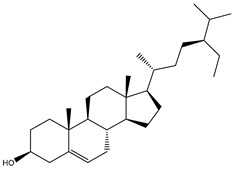	−10.3	MET49 (2.16 Å), GLN189 (2.95 Å)
Brassicasterol (222)	*D. coccinea*	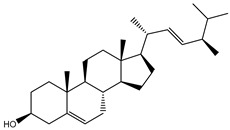	−10.1	MET49 (2.17 Å)
3β-Hexadecanoylcholest-5-en-7-one (202)	*A. dichotoma*	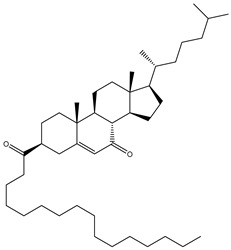	−10.0	GLY143 (1.95 Å)
Sipholenone E (163)	*S. siphonella*	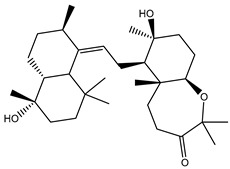	−9.9	GLN189 (1.83 Å)

^a^ Only hydrogen bonds (in Å) were listed. ^b^ No plant source was noticed.

**Table 2:** Components of the MM/GBSA binding energies for erylosides B (226)- and lopinavir-M^pro^ complexes as determined by MD simulation at 100 ns.

Compound Name	Calculated MM/GBSA Binding Energy (kcal/mol)						
	∆*E*_VDW_^a^	∆*E*_ele_^b^	∆*E*_GB_^c^	∆*E*_SUR_^d^	∆*G*_gas_^e^	∆*G*_Solv_^f^	∆*G*_binding_^g^
Erylosides B (226)	−71.2	−30.5	58.1	−8.3	−101.7	49.8	−51.9
Lopinavir	−45.6	−22.1	39.9	−5.7	−67.8	34.2	−33.6

^a^ van der Waals energy. ^b^ electrostatic energy. ^c^ The electrostatic solvation free energy calculated from the generalized Born equation. ^d^ The nonpolar component of the solvation energy. ^e^ Total gas phase energy. ^f^ The solvation free energy. ^g^ The evaluated free energy calculated from the terms above.

**Table 3:** Distance, occupancy, and hydrogen bonding for erylosides B (226) and lopinavir with key M^pro^ amino acid residues.

Compound Name	Acceptor	Donor	Distance (Å) ^a^	Angle (degree) ^a^	Occupied (%) ^b^
Erylosides B (226)	GLN_189@O	Erylosides B @O5-H29	2.9	142	95.7
	GLU166@O	Erylosides B @O3-H16	2.8	141	92.3
	CYS145@O	Erylosides B @O12-H44	2.9	152	91.1
	ASN142@O	Erylosides B @O16-H29	2.7	156	83.3
Lopinavir	GLN189@O	Lopinavir @O9-H19	2.8	145	85.6
	GLY143@O	Lopinavir @O12-H28	2.7	158	75.6

^a^ The hydrogen bonds are inspected by the acceptor-H-donor angle of >120° and acceptor-donor atom distance < 3.5 Å. ^b^ Occupancy is employed to estimate the strength and stability of the hydrogen bond.

## Data Availability

Data sharing is not applicable to this article.

## Supplementary Materials

The following are available online. Figure S1: 2D representations of interactions of lopinavir and the top 27 potent marine natural products (MNPs) with the proximal amino acid residues of SARS-CoV-2 main protease (M^pro^), Figure S2: 3D representations of predicted binding modes of (i) 190, (ii) 178, (iii) 226 and (iv) lopinavir towards SARS-CoV-2 main protease (M^pro^), Figure S3: 3D representations of binding modes of (i) erylosides B (226)- and (ii) lopinavir-M^pro^ complexes according to an average structure over a 100 ns MD simulation, Figure S4: a genome-wide Reactome hierarchy map of the pathways influenced by the top 20 gene targets in response to erylosides B (226) in term of SARS-CoV-2 infection, Table S1: evaluated docking score (in kcal/mol) for lopinavir and all investigated marine natural products (MNPs) against SARS-CoV-2 main protease (M^pro^), Table S2: computed Autodock and MM/GBSA binding energies (in kcal/mol) for the top 27 potent marine natural products (MNPs) against SARS-CoV-2 main protease (M^pro^) over 250 ps implicit solvent MD simulations, Table S3: network topological analysis for the predicted targets for erylosides B (226), Table S4: top 20 most relevant pathways for erylosides B (226) targets resulted from Pathway Enrichment Analysis (PEA).

## Sample Availability

The samples are not available from the authors.
