# Supplementary material for: In Silico Mining of Terpenes from Red-Sea Invertebrates for SARS-CoV-2 Main Protease (Mpro) Inhibitors
Source: Molecules. 2021 Apr 5;26(7):2082. doi: 10.3390/molecules26072082 (PMC8038614; doi:10.3390/molecules26072082)
Supplement: Supplementary file 1 [file molecules-26-02082-s001.pdf]

molecules

MDPI

Article

In Silico Mining of Terpenes from Red-Sea Invertebrates for SARS-CoV-2 Main Protease (M^{pro}) Inhibitors

Mahmoud A. A. Ibrahim [1,*], Alaa H. M. Abdelrahman [1], Tarik A. Mohamed [2], Mohamed A. M. Atia [3], Montaser A. M. Al-Hammady [4], Khlood A. A. Abdeljawaad [1], Eman M. Elkady [4], Mahmoud F. Moustafa [5,6], Faris Alrumaihi [7], Khaled S. Allemailem [7], Hesham R. El-Seedi [8,9,10,*], Paul W. Paré [11], Thomas Efferth [12] and Mohamed-Elamir F. Hegazy [2,12,*]

1 Computational Chemistry Laboratory, Chemistry Department, Faculty of Science, Minia University, Minia 61519, Egypt; a.abdelrahman@compchem.net (A.H.M.A.); kh.abdeljawaad@compchem.net (K.A.A.A.)
2 Chemistry of Medicinal Plants Department, National Research Centre, 33 El-Bohouth St., Dokki, Giza 12622, Egypt; tarik.nrc83@yahoo.com
3 Molecular Genetics and Genome Mapping Laboratory, Genome Mapping Department, Agricultural Genetic Engineering Research Institute (AGERI), Agricultural Research Center (ARC), Giza 12619, Egypt; matia@ageri.sci.eg
4 National Institute of Oceanography & Fisheries, NIOF, 11516 Cairo, Egypt; coralreef_noif1@yahoo.com (M.A.M.A-H); emelkady@yahoo.com (E.M.E.).
5 Department of Biology, College of Science, King Khalid University, Abha 9004, Saudi Arabia; hamdony@yahoo.com
6 Department of Botany & Microbiology, Faculty of Science, South Valley University, Qena, Egypt
7 Department of Medical Laboratories, College of Applied Medical Sciences, Qassim University, Buraydah, Saudi Arabia; f_alrumaihi@qu.edu.sa (F.A.); k.allemailem@qu.edu.sa (K.S.A.)
8 Department of Molecular Biosciences, The Wenner-Gren Institute, Stockholm University, S-106 91 Stockholm, Sweden; hesham.elseedi@su.se
9 Department of Chemistry, Faculty of Science, El-Menoufia University, Shebin El-Kom 32512, Egypt
10 International Research Center for Food Nutrition and Safety, Jiangsu University, Zhenjiang 212013, China
11 Department of Chemistry & Biochemistry Texas Tech University, Lubbock, TX 79409 USA; paul.pare@ttu.edu
12 Department of Pharmaceutical Biology, Institute of Pharmaceutical and Biomedical Sciences, Johannes Gutenberg University, Staudinger Weg 5, 55128 Mainz, Germany; efferth@uni-mainz.de
* Correspondence: m.ibrahim@compchem.net (M.A.A.I.); hesham.elseedi@su.se (H.R.E.-S.); elamir77@live.com (M.E.F.H.); Tel.: +2-010-241-61-444 (M.A.A.I.); +46-73-566-8234 (H.R.E.−S.); +2-033-371-635 (M.E.F.H.)

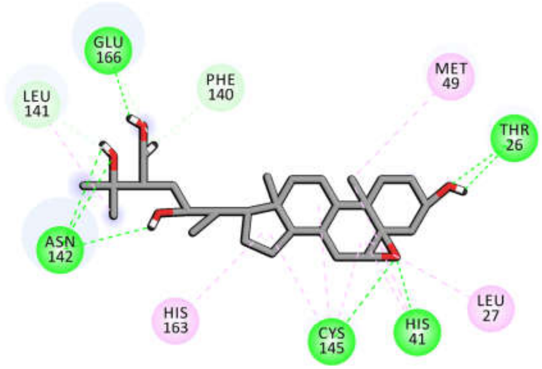

190

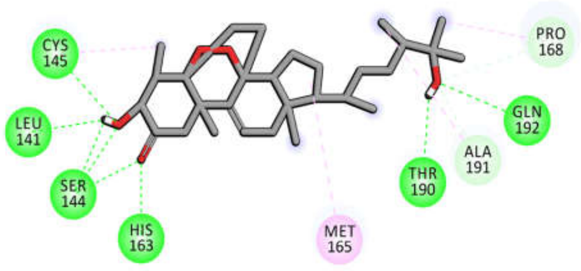

178

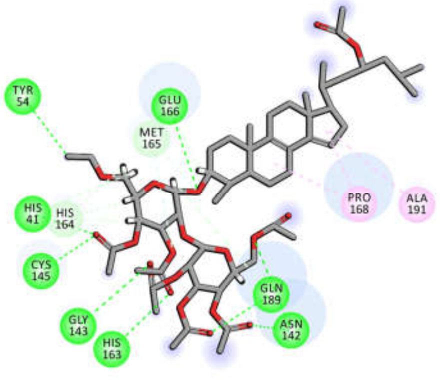

226

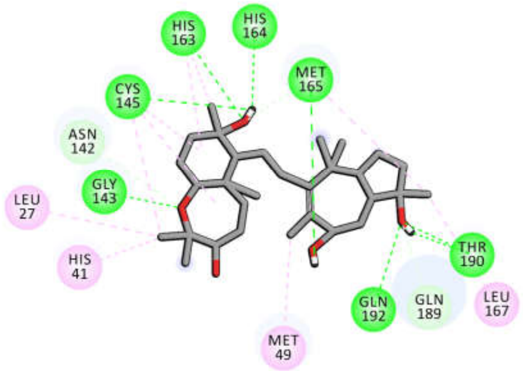

157

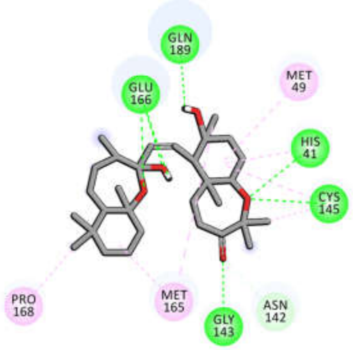

162

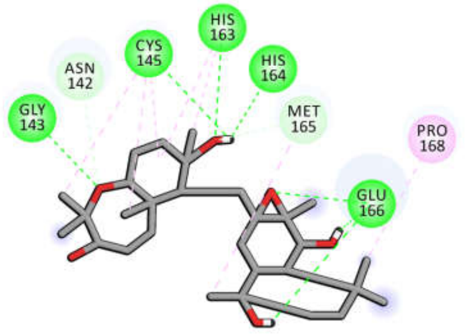

174

Figure S1. 2D representations of interactions of lopinavir and the top 27 potent marine natural products (MNPs) with the proximal amino acid residues of SARS-CoV-2 main protease (M^{pro}).

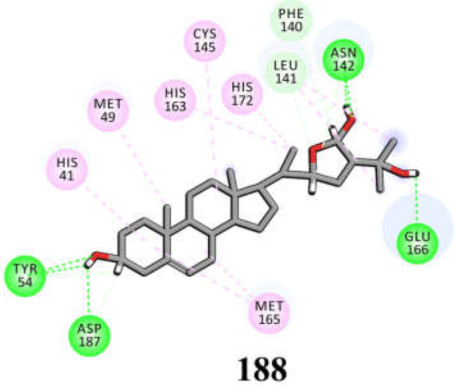

188

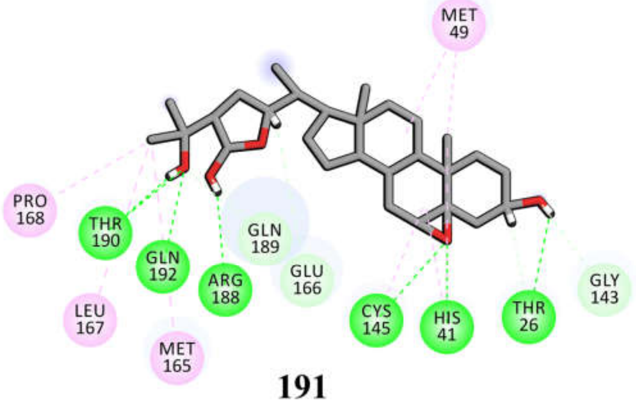

191

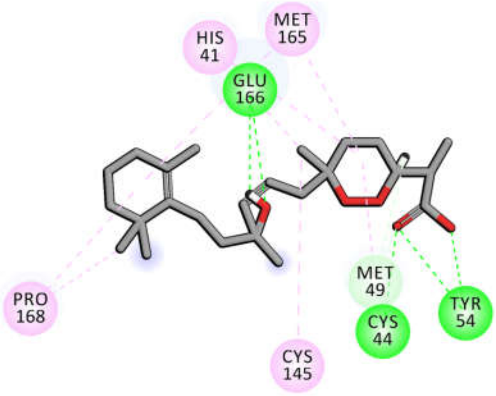

144

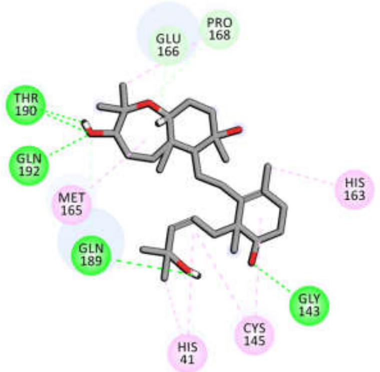

172

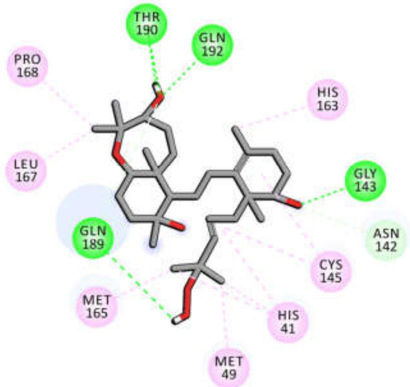

171

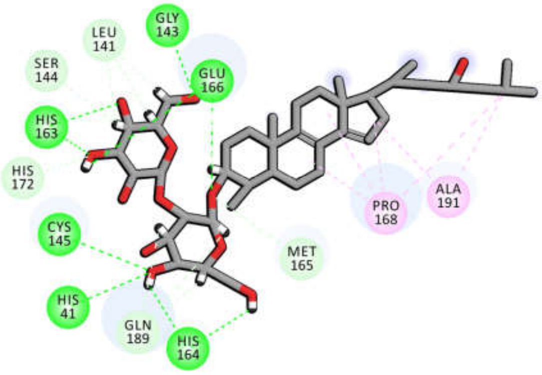

224

Figure S1. Continued.

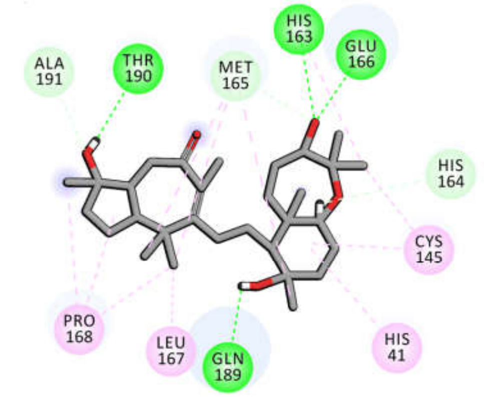

176

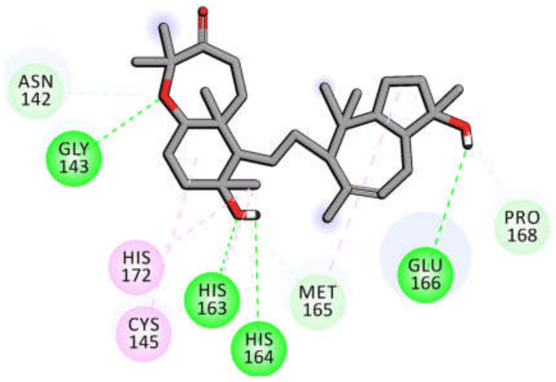

175

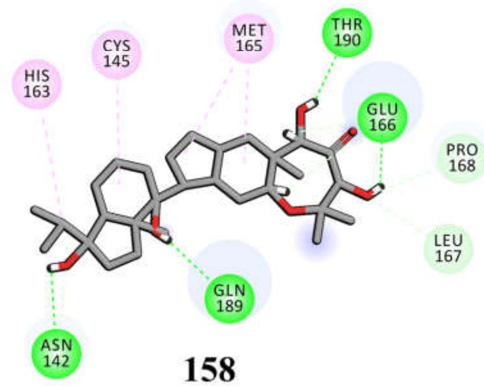

158

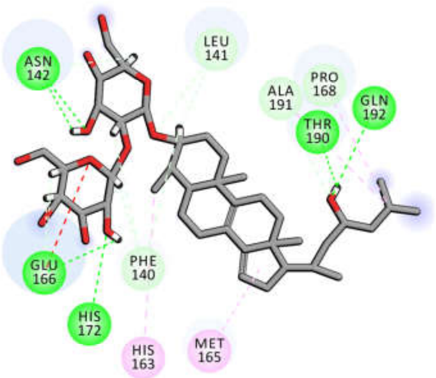

197

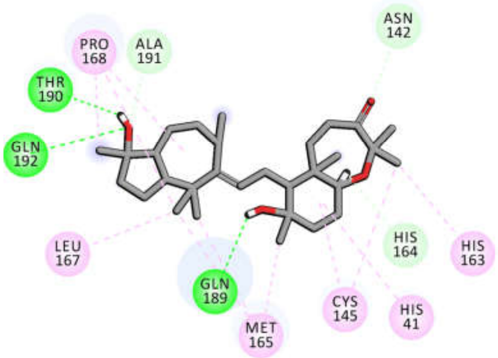

155

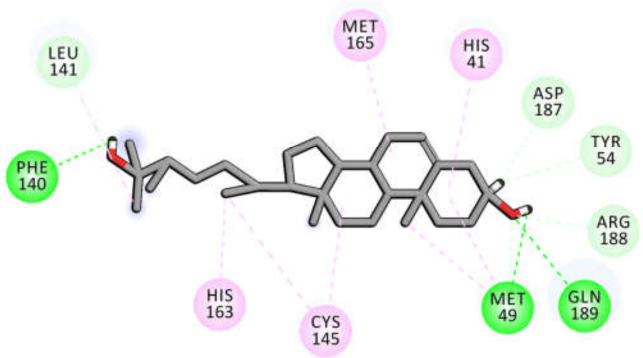

187

Figure S1. Continued.

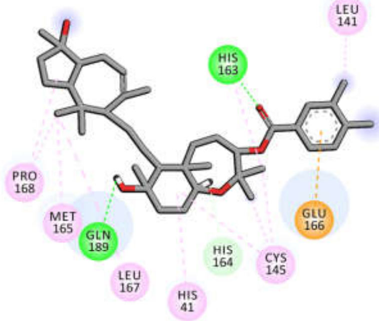

151

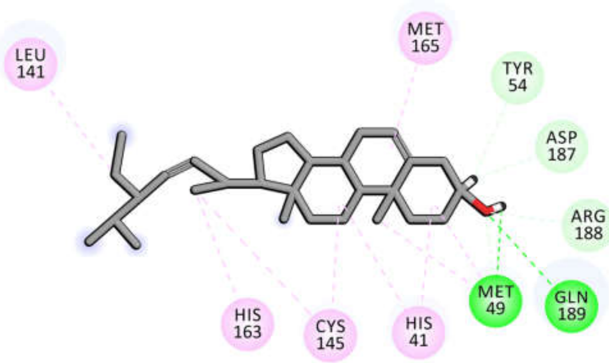

220

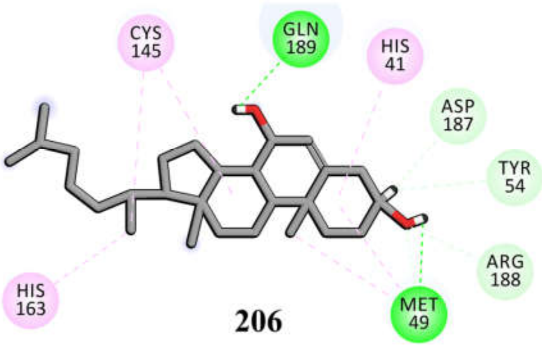

206

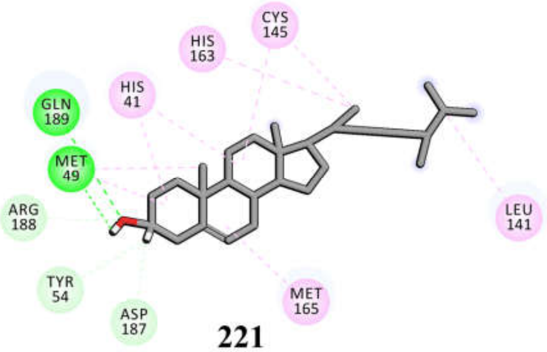

221

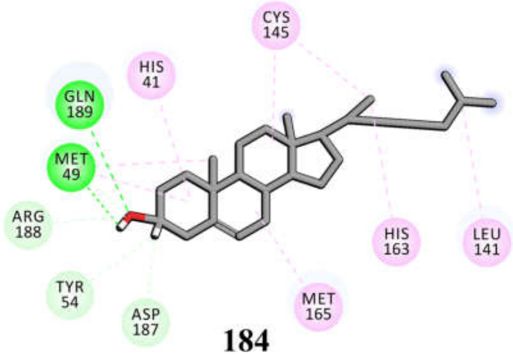

184

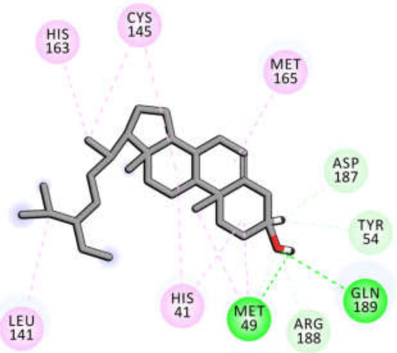

219

Figure S1. Continued.

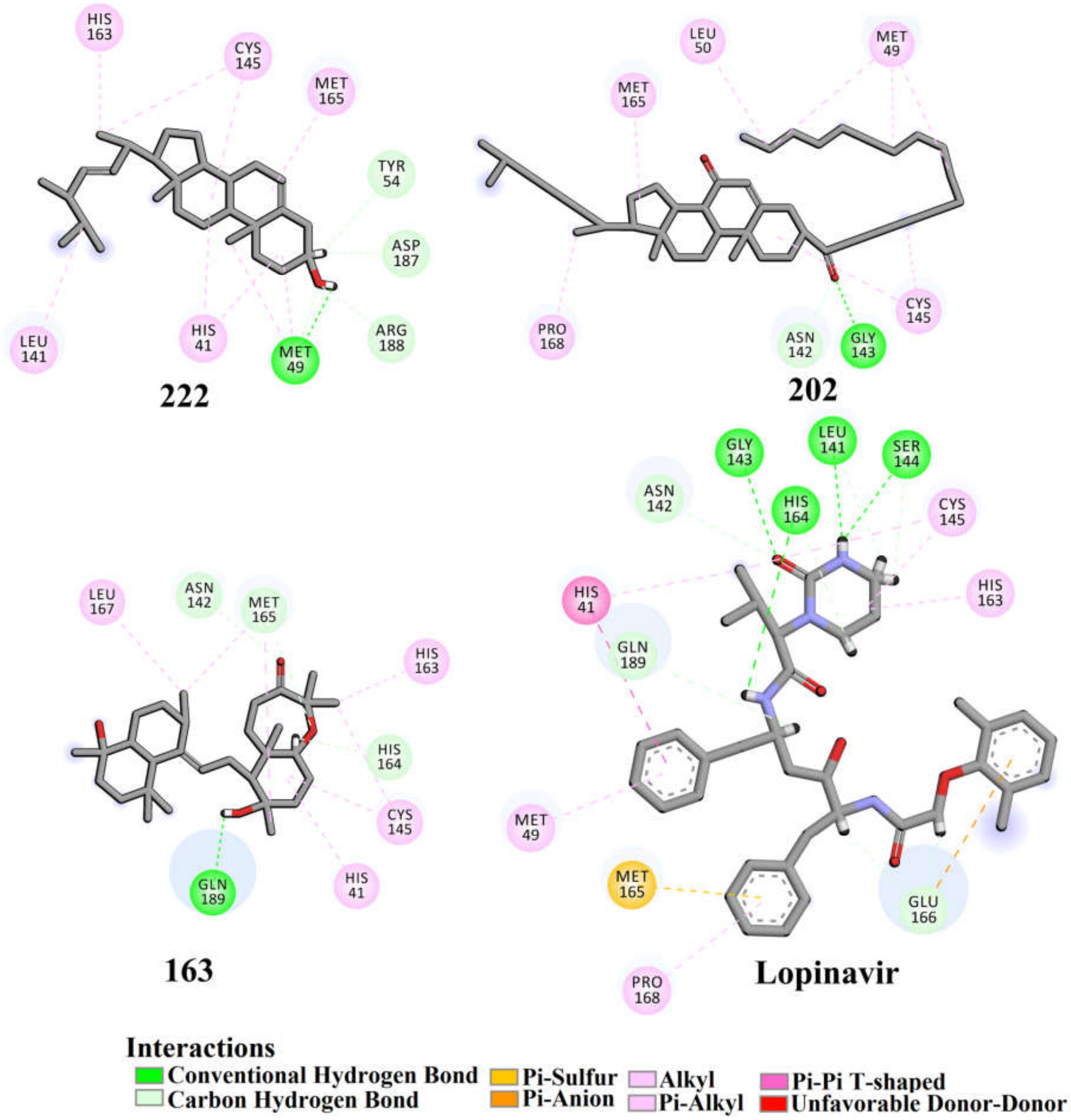

Figure S1. Continued.

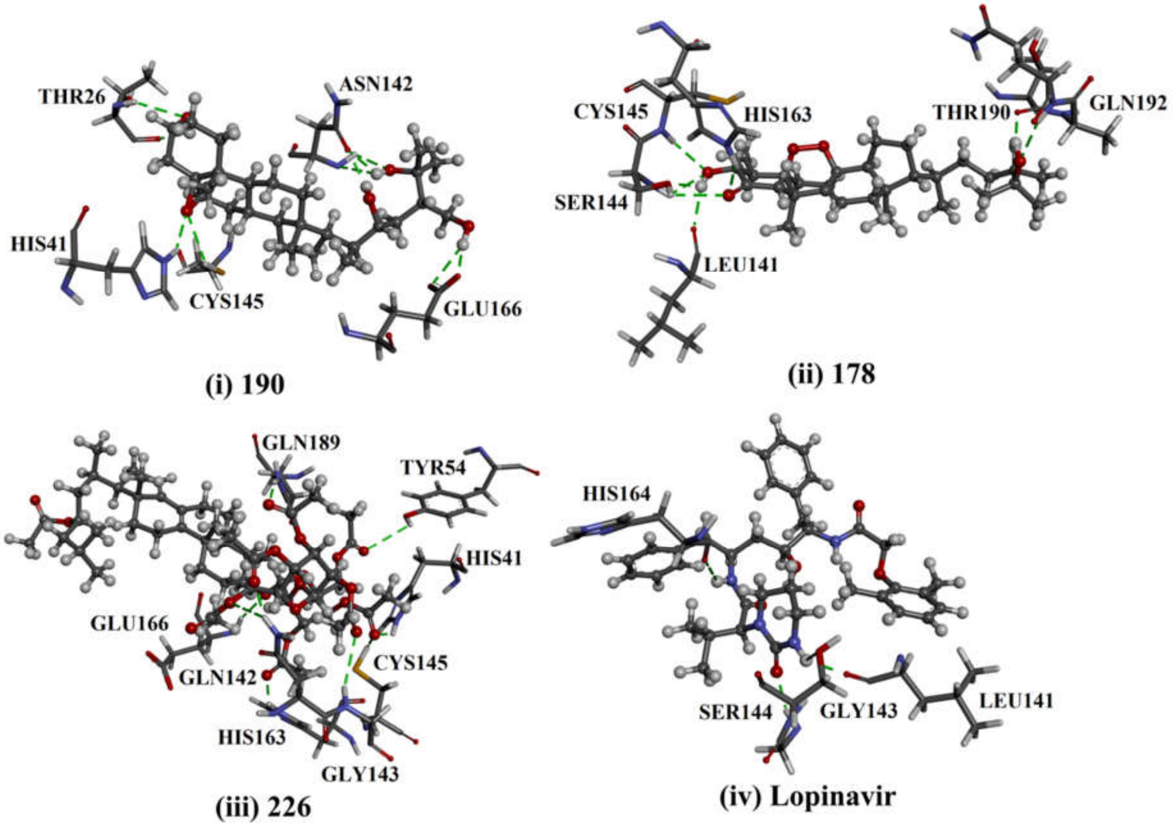

Figure S2. 3D representations of predicted binding modes of (i) **190**, (ii) **178**, (iii) **226** and (iv) lopinavir towards SARS-CoV-2 main protease (M^{pro}).

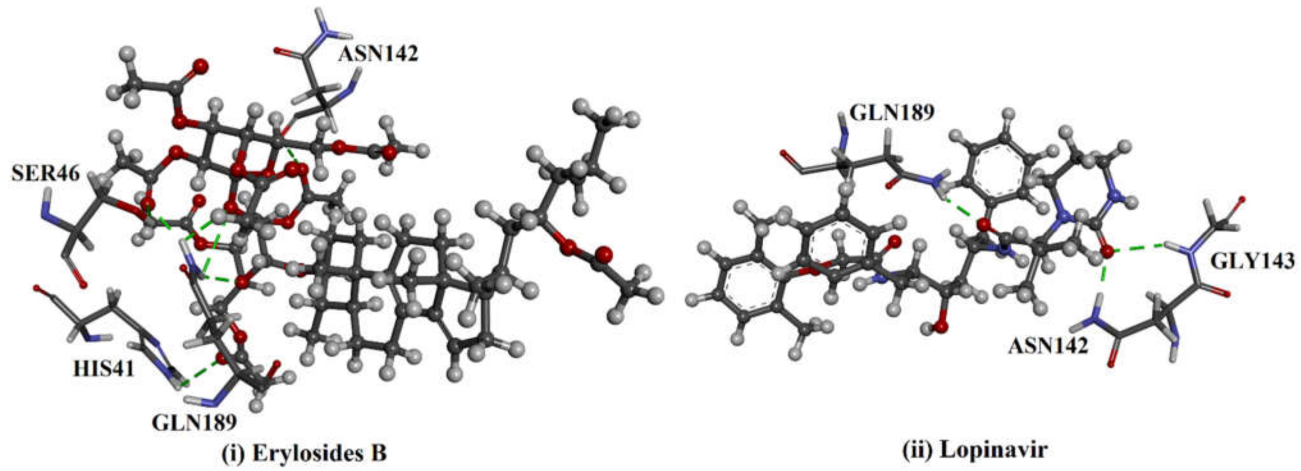

Figure S3. 3D representations of binding modes of (i) erylosides B (**226**)- and (ii) lopinavir-M^{pro} complexes according to an average structure over a 100 ns MD simulation.

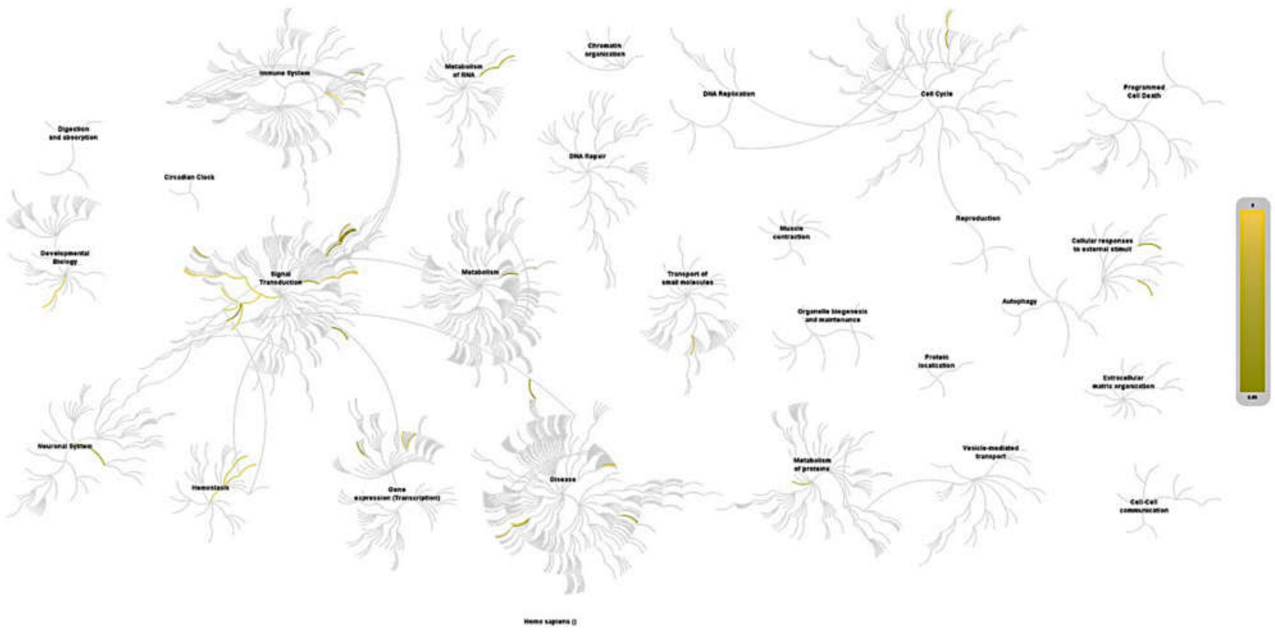

Figure S4. A genome-wide Reactome hierarchy map of the pathways influenced by the top 20 gene targets in response to erylosides B (**226**) in term of SARS-CoV-2 infection. Reactome pathways are arranged in a hierarchy. Each step away from the Center represents the next level lower in the pathway hierarchy. The color code denotes the over-representation of that pathway in the input dataset. Light grey signifies pathways that are not significantly over-represented.

Table S1. Evaluated docking score (in kcal/mol) for lopinavir and all investigated marine natural products (MNPs) against SARS-CoV-2 main protease (M^pro^).

No.	Compound Name	Plant Source	Chemical Structure	Docking Score (kcal/mol)
1	Lopinavir	---	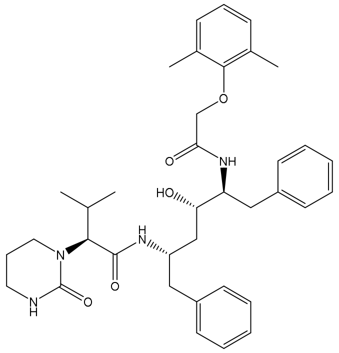	−9.8
2	Depresosterol **(190)**	*L. depressum*	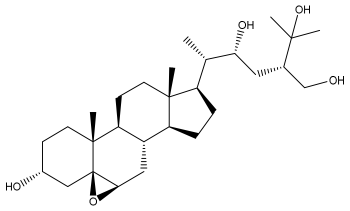	−12.3
3	3β-25-Dihydroxy-4-methyl-5α,8α-epidioxy-2-ketoergost-9-ene **(178)**	*Sinularia candidula*	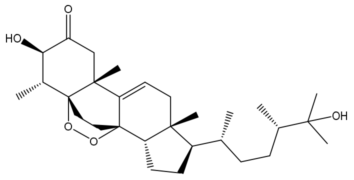	−12.2
4	Erylosides B **(226)**	*E. lendenfeldi*	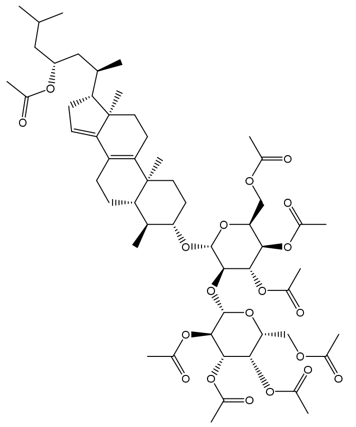	−12.1
5	Sipholenol H **(157)**	*S. siphonella*	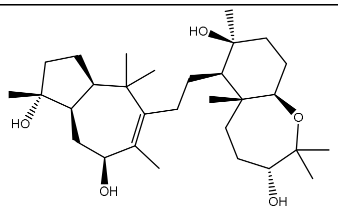	−12.0

6	Dahabinone A **(162)**	*S. siphonella*	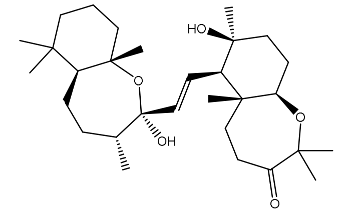	−11.9
7	Sipholenol I **(174)**	*S. siphonella*	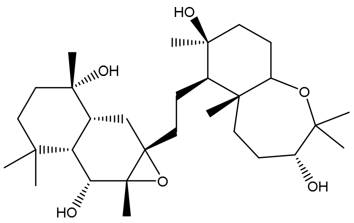	−11.8
8	Lobophytosterol **(188)**	*L. depressum*	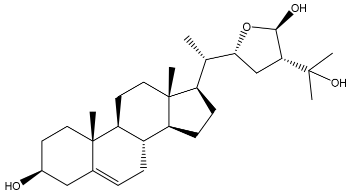	−11.5
9	(22R,24E,28E)-5β,6β-Epoxy-22,28-oxido-24-methyl-5αcholestan-3β,25,28-triol **(191)**	*L. depressum*	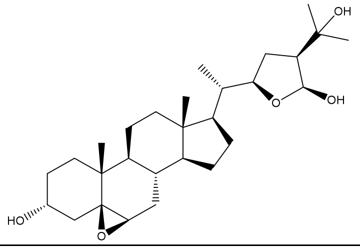	−11.4
10	Tasnemoxide A **(144)**	*D. erythraeanus*	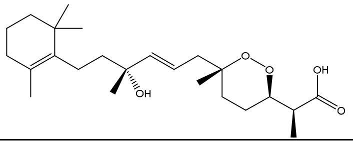	−11.4
11	Siphonellinol C **(172)**	*S. siphonella*	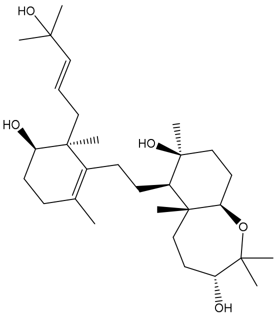	−11.3

12	Siphonellinol-C-23-hydroperoxide **(171)**	*S. siphonella*	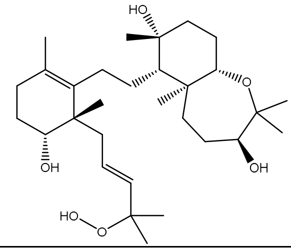	−11.2
13	Erylosides K **(224)**	*Erylus lendenfeldi*	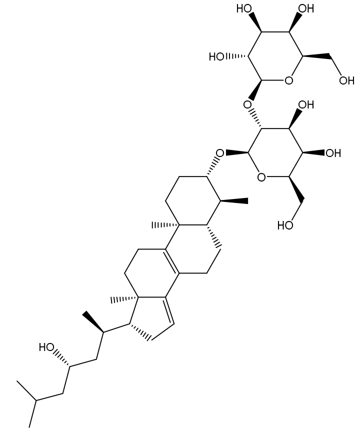	−11.1
14	Sipholenol D **(176)**	*S. siphonella*	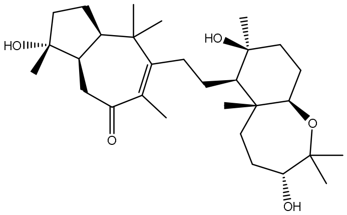	−11.0
15	Sipholenone A **(175)**	*S. siphonella*	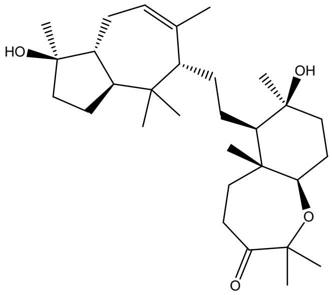	−11.0
16	Neviotine B **(158)**	*S. siphonella*	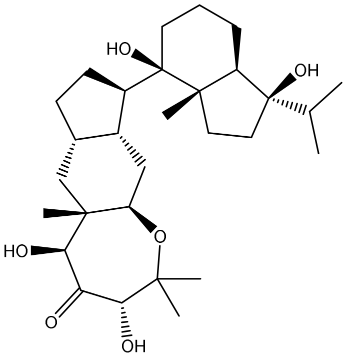	−10.9

17	Eryloside A **(197)**	*Genus Erylus*	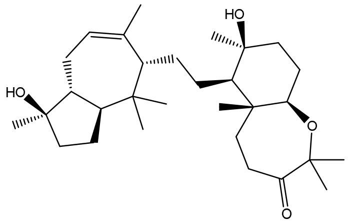	−10.7
18	Sipholenone D **(155)**	*S. siphonella*	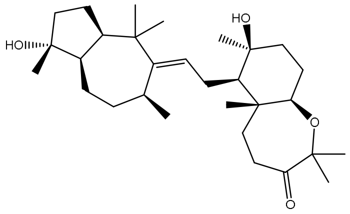	−10.7
19	24-Methylcholestane-5-en-3β,25-diol **(187)**	*S. polydactyla*	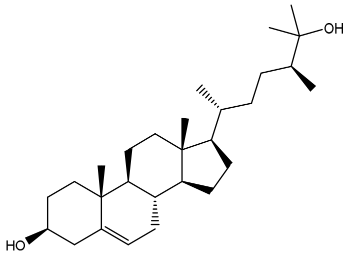	−10.6
20	SipholenolA-4-O-3′,4′-dichlorobenzoate **(151)**	*S. siphonella*	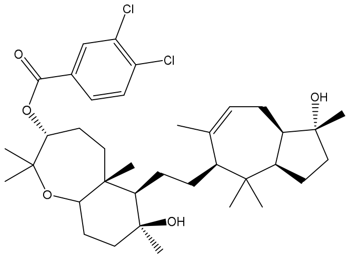	−10.5
21	Stigmasterol **(220)**	*D. coccinea*	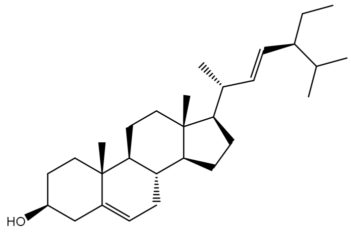	−10.5
22	Cholest-5-en-3β,7β-diol **(206)**	*A. dichotoma*	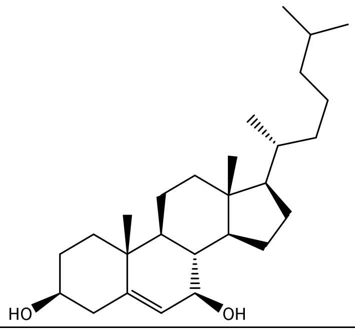	−10.3

23	Campesterol **(221)**	*D. coccinea*	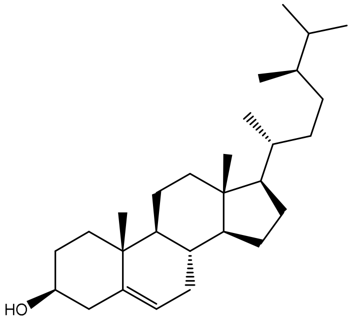	−10.3
24	Cholesterol **(184)**	*Dendronephthya*	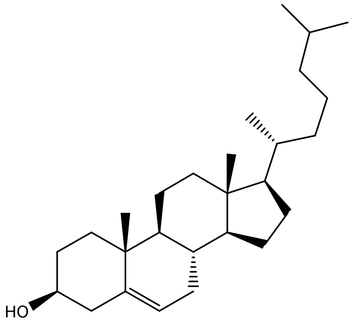	−10.3
25	Clionasterol **(219)**	*Dragmacidon coccinea*	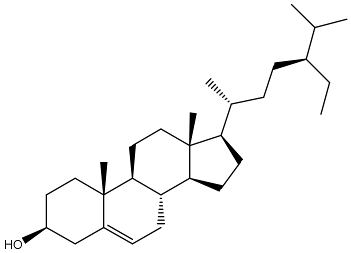	−10.3
26	Brassicasterol **(222)**	*D. coccinea*	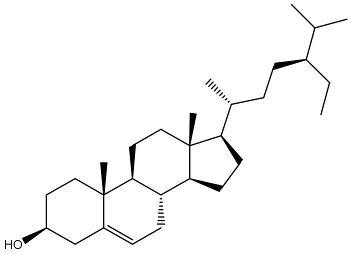	−10.1
27	3β-Hexadecanoylcholest-5-en-7-one **(202)**	*A. dichotoma*	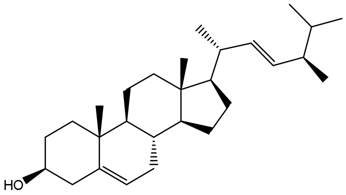	−10.0

28	Sipholenone E **(163)**	*S. siphonella*	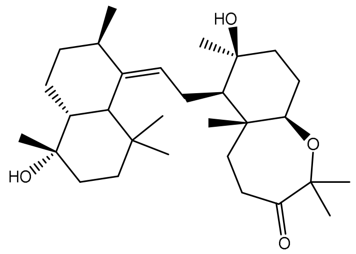	−9.9
29	β-Sitosterol-3-O-(3Z)-pentacosenoate **(183)**	*E. gibbosa*	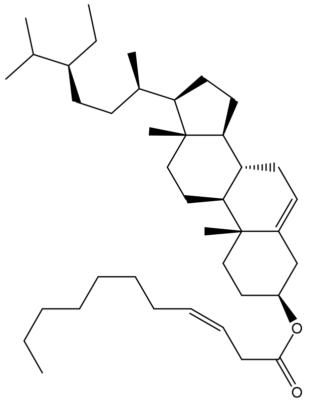	−9.8
30	3β,7α-Dihydroxy-cholest-5-ene **(199)**	*A. dichotoma*	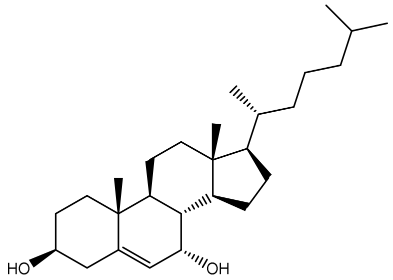	−9.7
31	Cholest-5-en-7β-methyl-3β-yl formate **(209)**	*Petrosia sp.*	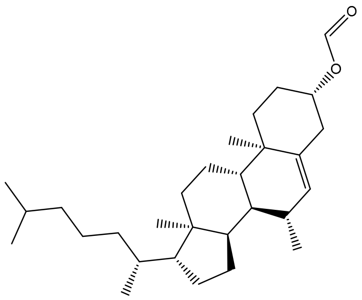	−9.7
32	Juncins A **(99)**	*J. juncea*	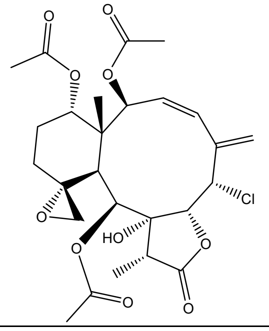	−9.7

33	Sigmosceptrellin B **(138)**	*D. erythraeanus*	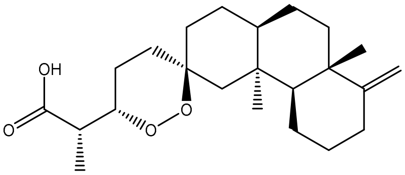	−9.7
34	16-epi-Scalarolbutenolide **(117)**	*H. erecta*	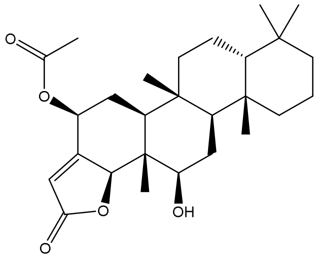	−9.6
35	25-Dehydroxy-12-epi-deacetylscalarin **(115)**	*Hyrtios erecta*	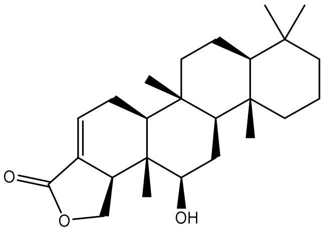	−9.6
36	7-Dehydrocholesterol **(211)**	*Petrosia sp.*	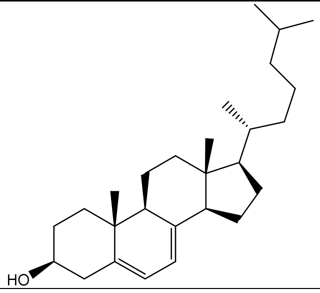	−9.6
37	Dendronesterone A **(185)**	*Dendronephthya*	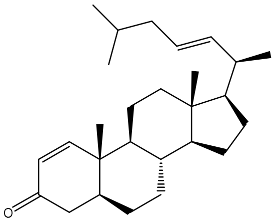	−9.5
38	Salmahyrtisol B **(121)**	*H. erecta*	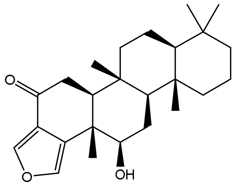	−9.5

39	Sipholenol G **(154)**	*S. siphonella*	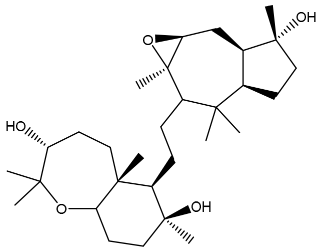	−9.5
40	Sigmosceptrellin B methyl ester **(139)**	*D. erythraeanus*	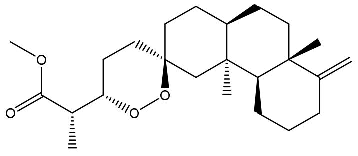	−9.5
41	3β-Hydroxycholest-5-en-7-one **(205)**	*A. dichotoma*	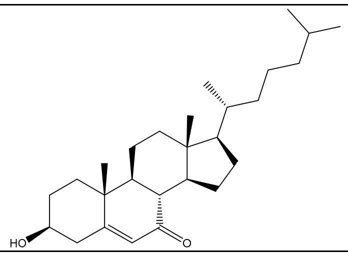	−9.5
42	Nuapapuin A methyl ester **(132)**	*Diacarnus erythraeanus*	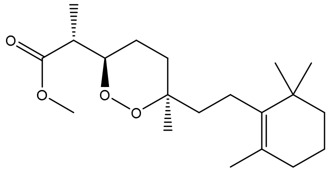	−9.4
43	Sipholenol A **(150)**	*S. siphonella*	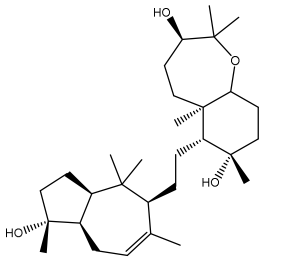	−9.4
44	Siphonellinol E **(170)**	*S. siphonella*	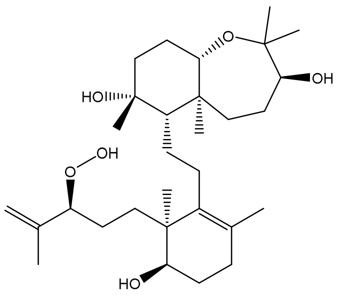	−9.4

45	Sipholenol F **(156)**	*S. siphonella*	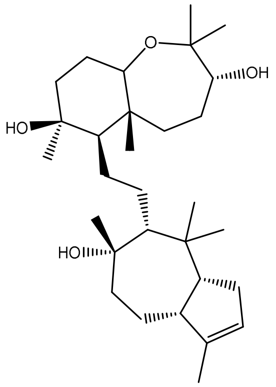	−9.4
46	Sipholenol L **(164)**	*S. siphonella*	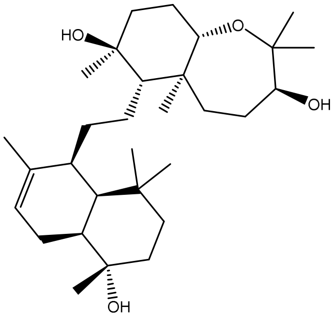	−9.4
47	Norrisolide **(110)**	*Dysidea sp.*	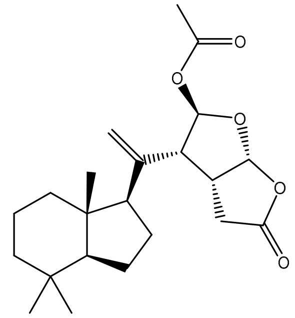	−9.3
48	12-O-Deacetyl-12-epi-scalarine **(126)**	*H. erecta*	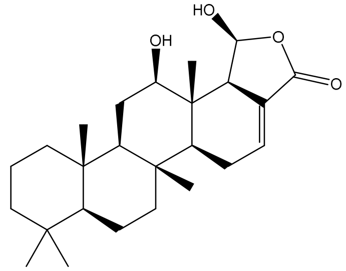	−9.3
49	Sinularolide C diacetate **(62)**	*L. crassum*	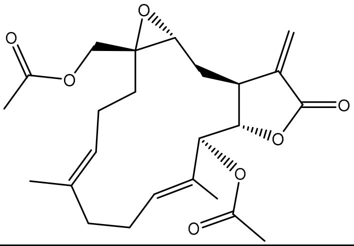	−9.3

50	(+)-Wistarin **(128)**	*I. wistarii*	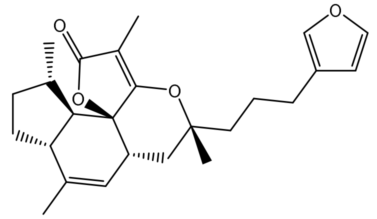	−9.3
51	epi-Sipholenol **(173)**	*S. siphonella*	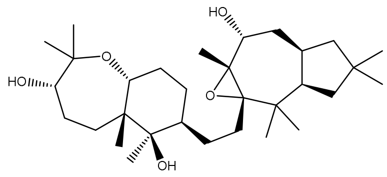	−9.3
52	Scalarolide **(123)**	*H. erecta*	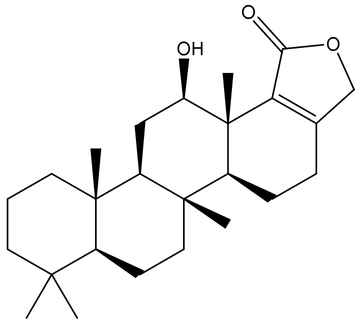	−9.2
53	(−)-Muqubilin A **(136)**	*D. erythraeanus*	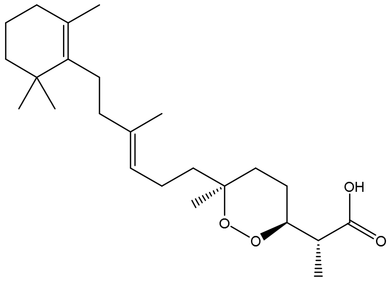	−9.2
54	Sesterstatin **(116)**	*H. erecta*	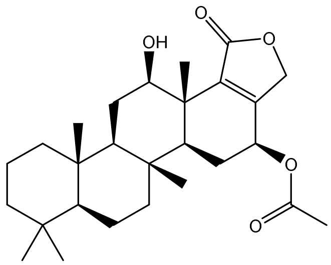	−9.2
55	Cholesta-8-en-3β,5α,6α,25-tetrol **(214)**	*Lamellodysidea*	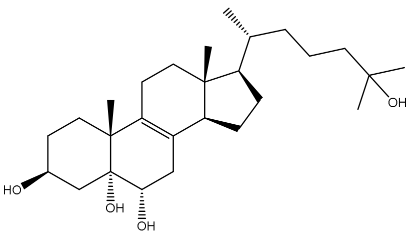	−9.1

56	7β-Acetoxy-24-methylcholesta-5-24(28)-diene-3,19-diol **(194)**	*L. arboreum*	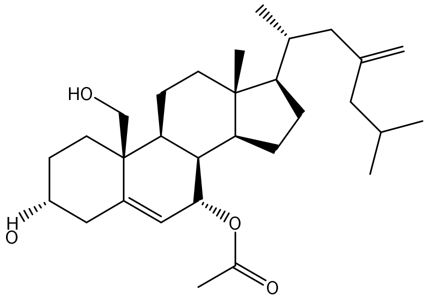	−9.1
57	Sipholenol J **(165)**	*S. siphonella*	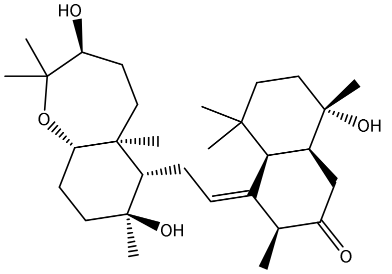	−9.1
58	Cholesta-8,24-dien-3β,5α,6α-triol **(216)**	*L. herbacea*	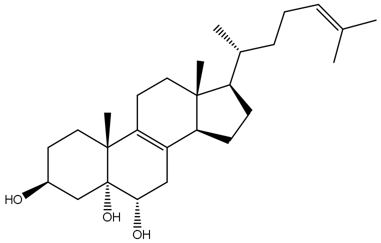	−9.0
59	Pachycladin A **(83)**	*Cladiella pachyclados*	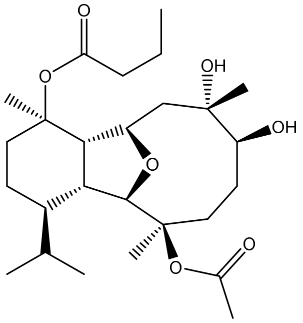	−9.0
60	16-Hydroxyscalarolide **(125)**	*H. erecta*	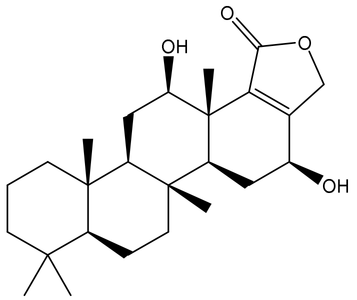	−9.0

61	(−)-9,10-Epoxymuqubilin A **(135)**	*D. erythraeanus*	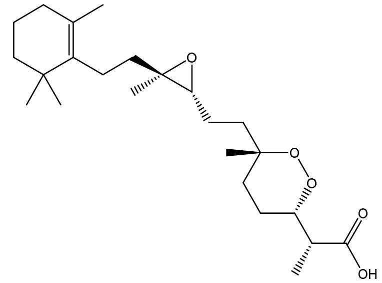	−9.0
62	Steroid	*Cystoseira trinode*	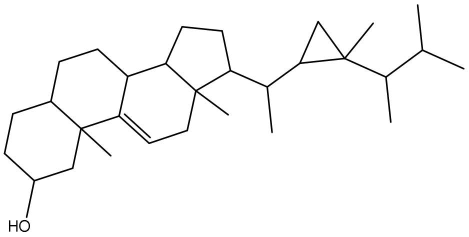	−8.9
63	(22E,24S)-5α,8α-Epidioxy-24 methylcholesta -6,22-dien-3β-ol **(200)**	*A. dichotoma*	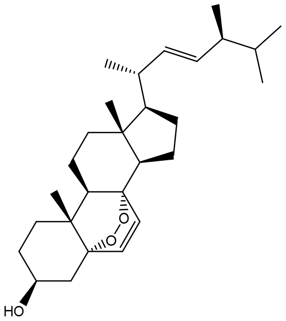	−8.9
64	5α-Pregna-3β-acetoxy-12β,16β-diol-20-one **(182)**	*Echinoclathria gibbosa*	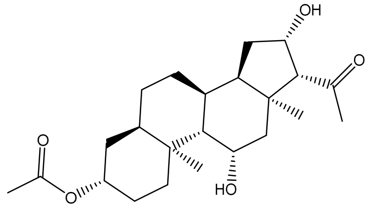	−8.9
65	Hyrtiosal **(120)**	*H. erecta*	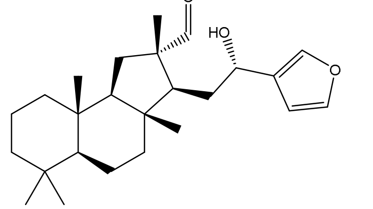	−8.9
66	(−)-13,14-Epoxymuqubilin A **(134)**	*D. erythraeanus*	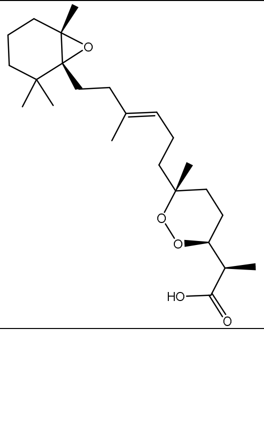	−8.9

67	24-Methylcholestane-3β,5α,6β,25-tetrol-25-monoacetate **(186)**	*Sinularia polydactyla*	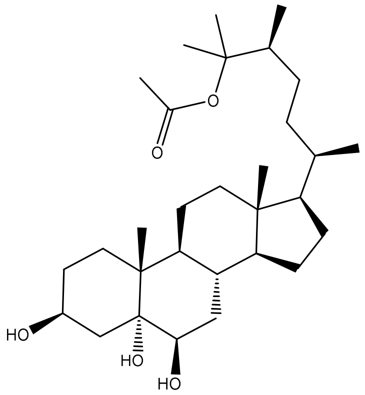	−8.9
68	7β-Chloro-8α-hydroxy-12-acetoxy-deepoxysarcophine **(49)**	*S. ehrenbergi*	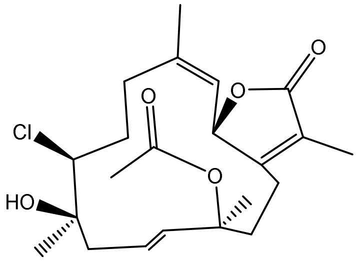	−8.9
69	(22E,24S)-5α,8α-Epidioxy-24-methylcholesta-6,9(11),22-trien-3β-ol **(201)**	*A. dichotoma*	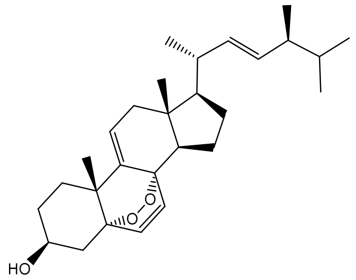	−8.9
70	3-Acetylsesterstatin **(118)**	*H. erecta*	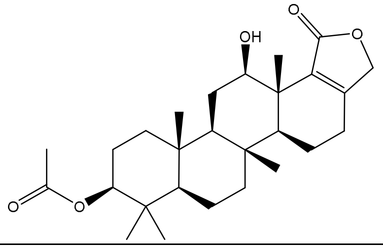	−8.9
71	19-Acetyl sesterstatin **(122)**	*H. erecta*	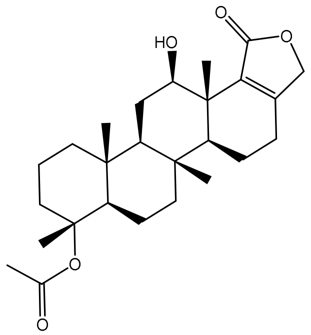	−8.9

72	Dendrotriol **(223)**	*Dendronephthya*	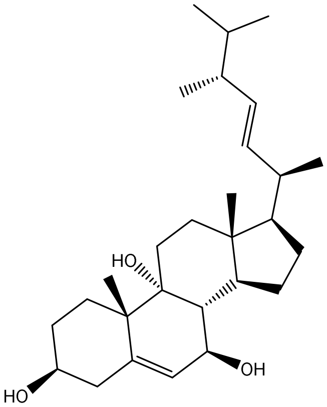	−8.8
73	Neviotine-A **(149)**	*Siphonochalina siphonella*	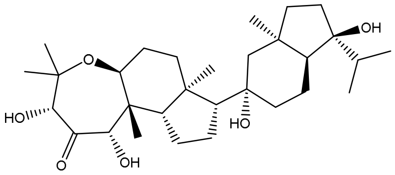	−8.8
74	24-Methylcholesta-5,24(28)-diene-3β-ol **(193)**	*Litophyton arboreum*	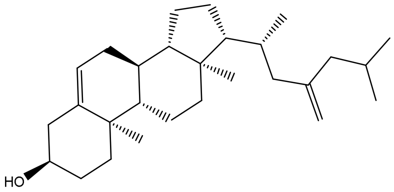	−8.8
75	(−)-Ircinianin **(129)**	*I. wistarii*	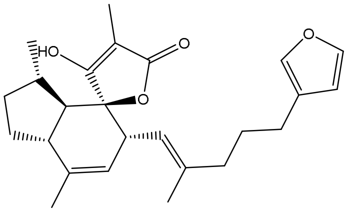	−8.8
76	Sipholenol M **(168)**	*S. siphonella*	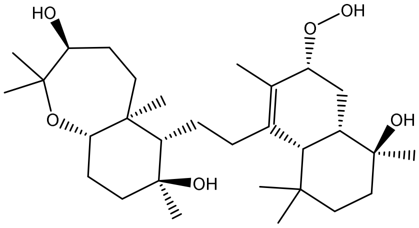	−8.8
77	3β,7β,9α-Trihydroxycholest-5-en **(208)**	*Petrosia*	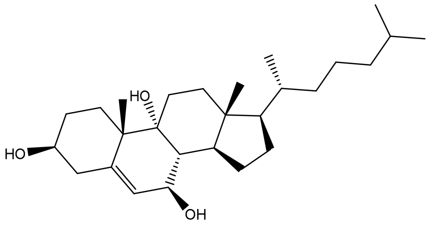	−8.8

78	Siphonellinol B **(161)**	*S. siphonella*	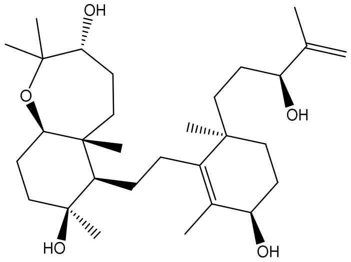	−8.8
79	1-hydroxy-1,4,4,6-tetramethyl-1,2,3,3a,4,5,8,8a-octahydroazulen-5-yl)-ethyl)-4a,6-dimethyloctahydro-2H-chromene-2,6-diol **(166)**	*S. siphonella*	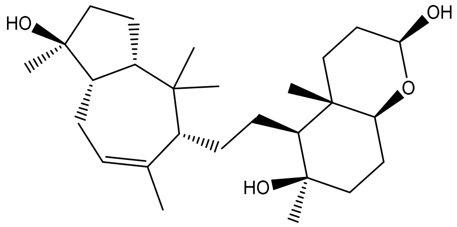	−8.8
80	5α,8α-Epidioxycholesta-6-en-3β-ol **(213)**	*Petrosia sp.*	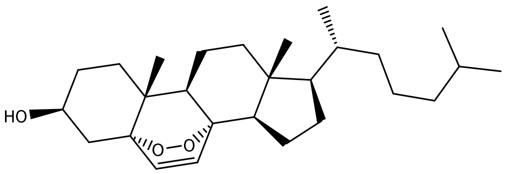	−8.8
81	(−)-Wistarin **(127)**	*Ircinia wistarii*	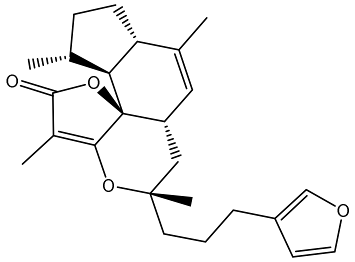	−8.7
82	Norsesterterpene	*Pocillopora verrucosa*	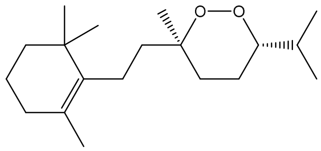	−8.7
83	5β,6β-Epoxy-24E-methylchloestan-3β,22(R),25-triol **(189)**	*L. depressum*	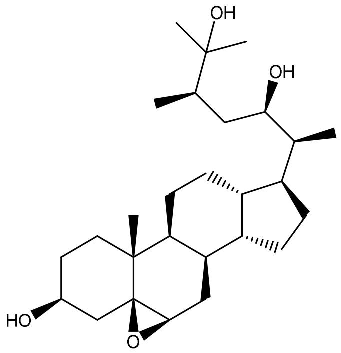	−8.7

84	22-Dehydrocholestrol **(207)**	*A. dichotoma*	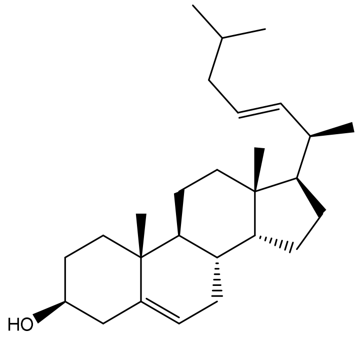	−8.7
85	24-Methylcholesta-5,24(28)-diene-3β,7β,19-triol **(195)**	*L. arboreum*	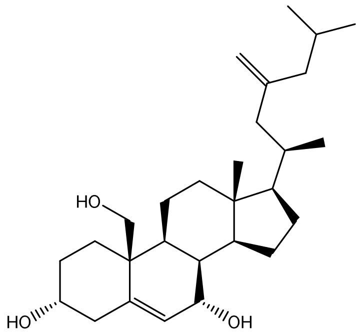	−8.7
86	Labolide **(58)**	*L. crassum*	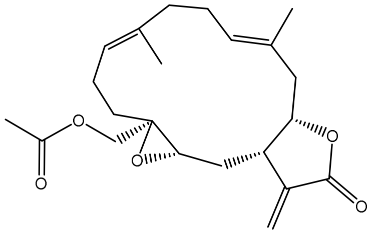	−8.7
87	Hyrtiosterol **(196)**	*Hyrtios Species*	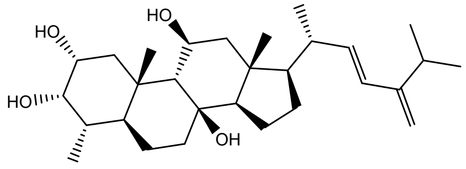	−8.6
88	Cholesta-8(14)-en-3β,5α,6α,25-tetrol **(215)**	*L. herbacea*	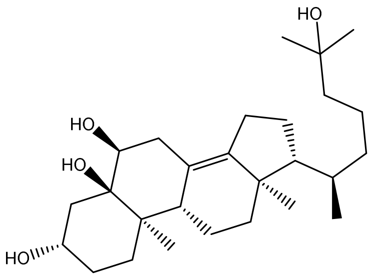	−8.6

89	Bilosespens A **(130)**	*Dysidea cinerea*	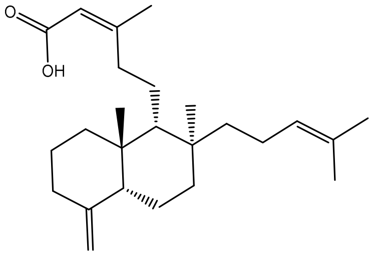	−8.6
90	Salmahyrtisol C **(124)**	*H. erecta*	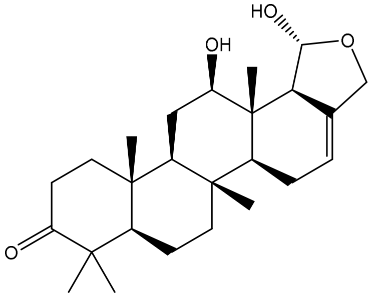	−8.6
91	5α,6α-Epoxycholest-8(14)-ene-3β,7α-diol **(212)**	*Petrosia sp.*	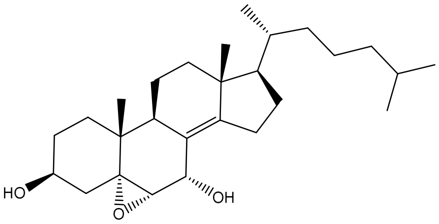	−8.6
92	Cholesta-8(14),24-dien-3β,5α,6α-triol **(217)**	*L. herbacea*	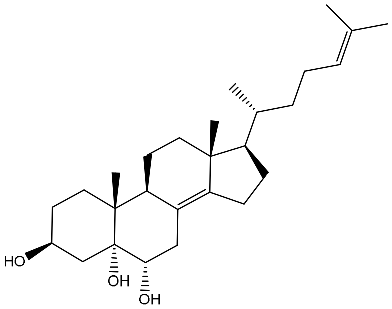	−8.6
93	xeniolide	*Acropora humilis*	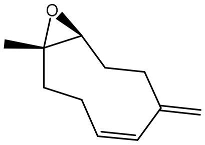	−8.6
94	Seco-norrlandin B **(112)**	*Dysidea sp.*	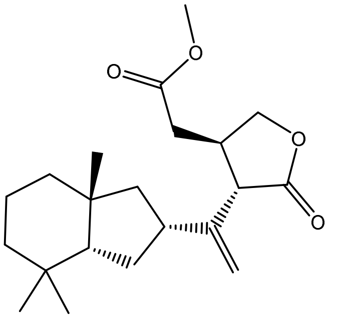	−8.5

95	3β-Hexadecanoylcholest-5-en-7-one **(202)**	*A. dichotoma*	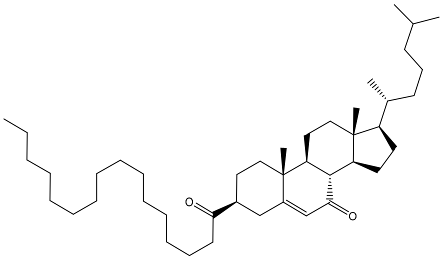	−8.5
96	Sesterterpene	*Pocillopora verrucosa*	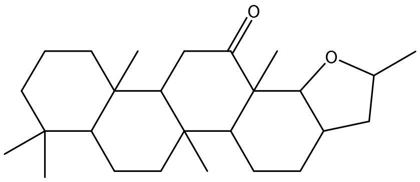	−8.5
97	Tasnemoxide B **(145)**	*D. erythraeanus*	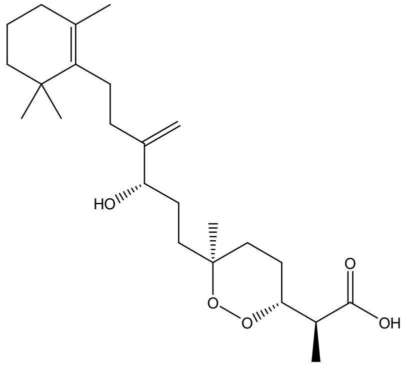	−8.5
98	Klysimplexin G **(84)**	*C. pachyclados*	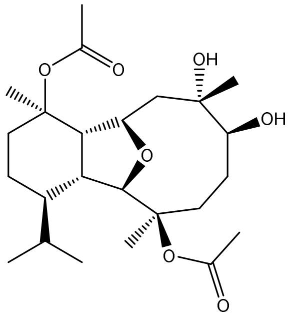	−8.5
99	Erylosides K **(224)**	*Erylus lendenfeldi*	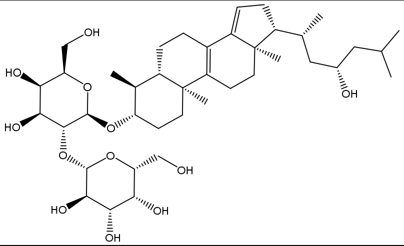	−8.5
100	(+)-Polyanthelin A **(96)**	*C. pachyclados*	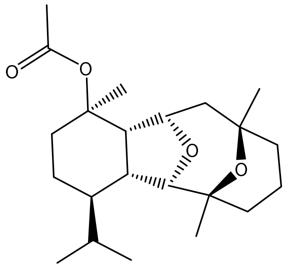	−8.5

101	Gorgosten-5(E)-3β-ol **(179)**	*Heteroxenia ghardaqensis*	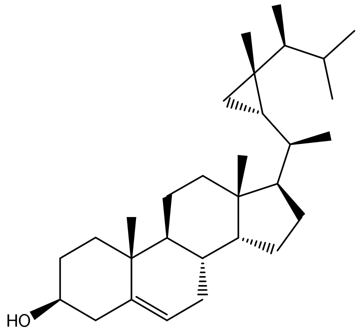	−8.5
102	Salmahyrtisol A **(119)**	*H. erecta*	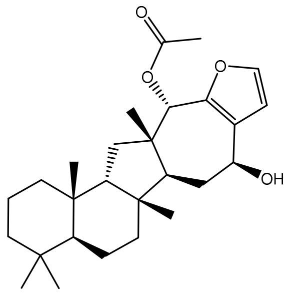	*−8.4*
103	3β-Hexadecanoylcholest-5-en-7β-ol **(203)**	*A. dichotoma*	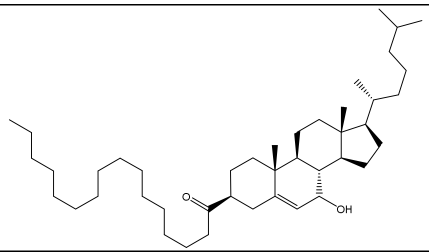	*−8.4*
104	Hurghaperoxide **(137)**	*D. erythraeanus*	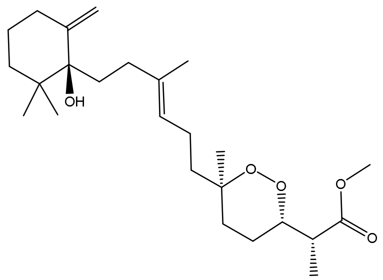	*−8.4*
105	Dehydroepiandrosterone **(210)**	*Petrosia sp.*	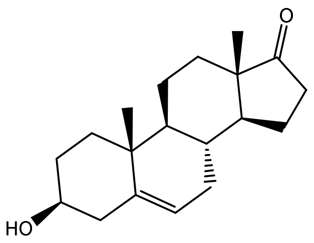	*−8.4*

106	(22R,24E)-24-Methylcholest-5-en-3β,22,25,28-tetraol **(192)**	*L. depressum*	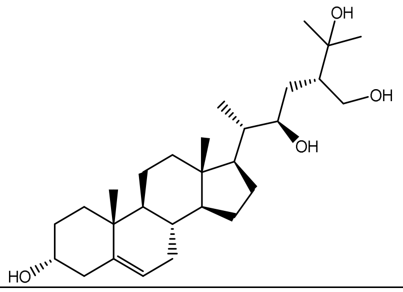	*−8.4*
107	Trochelioid A **(36)**	*S. trocheliophorum*	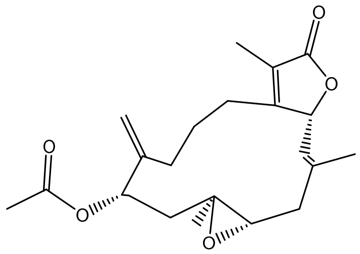	*−8.4*
108	Klysimplexin E **(86)**	*C. pachyclados*	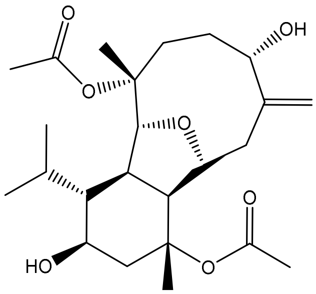	*−8.4*
109	Xenialactol-C **(75)**	*X. obscuronata*	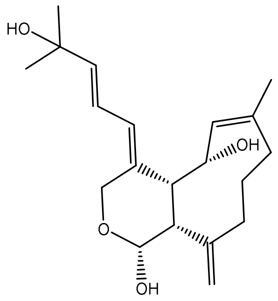	*−8.4*
110	7-Keto-8α-hydroxy-deepoxysarcophine **(48)**	*S. ehrenbergi*	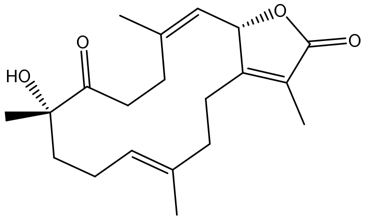	*−8.4*

111	Erylosides B **(226)**	*E. lendenfeldi*	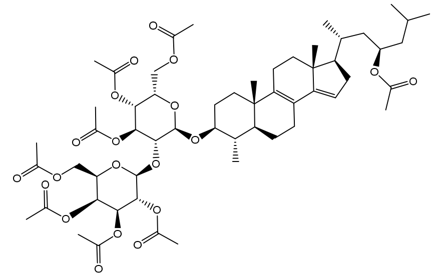	*−8.3*
112	Sipholenoside A **(159)**	*S. siphonella*	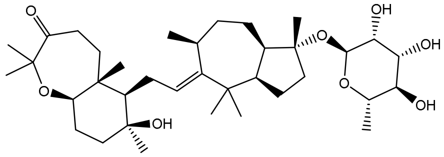	*−8.3*
113	Sipholenoside B **(160)**	*S. siphonella*	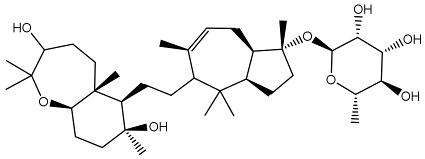	*−8.3*
114	Thunbergol **(57)**	*L. pauciflorum*	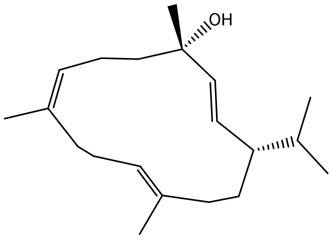	*−8.3*
115	14(15)-Epoxyxeniaphyllene **(77)**	*X. lilielae*	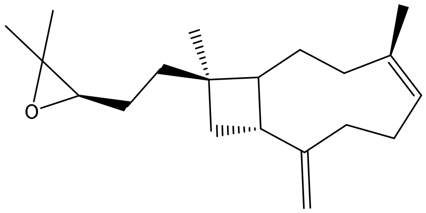	−8.2
116	Bilosespens A **(131)**	*D. cinerea*	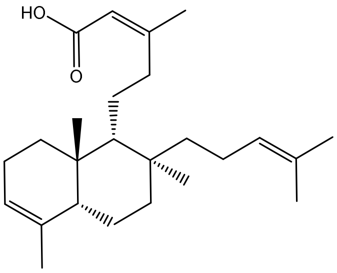	−8.2

117	Tasnemoxide C **(146)**	*D. erythraeanus*	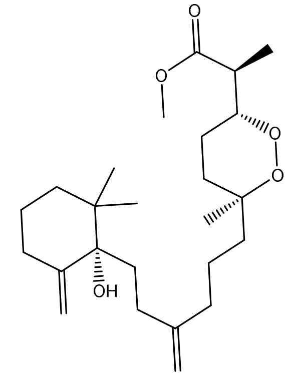	−8.2
118	Gorgostan-3β,5α,6β-triol-11α-acetate **(181)**	*H. ghardaqensis*	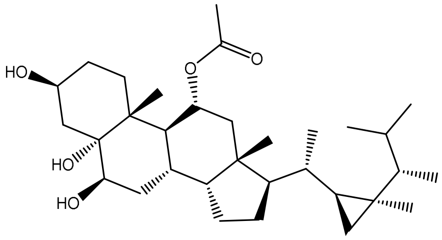	−8.2
119	Cembrane	*Nephthea molle*	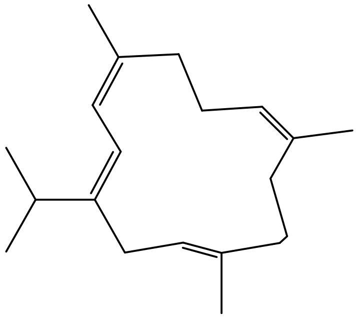	−8.2
120	Muqubilone **(148)**	*D. erythraeanus*	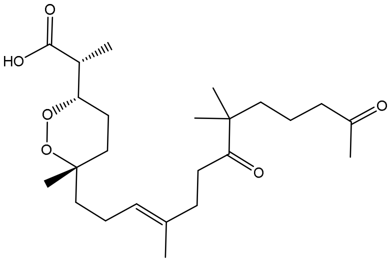	−8.2
121	Peyssonol A **(17)**	*Peyssonnelia sp.*	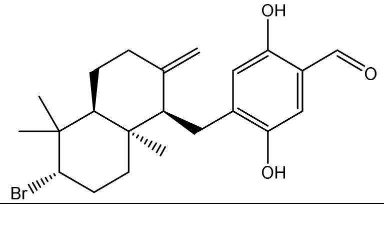	−8.1

122	l4,15-Xeniaphyllandiol-4,5-epoxide **(81)**	*X. macrosoiculata*	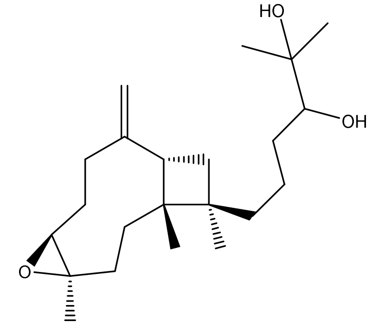	−8.1
123	Methyl-2-epinuapapuanoate **(133)**	*D. erythraeanus*	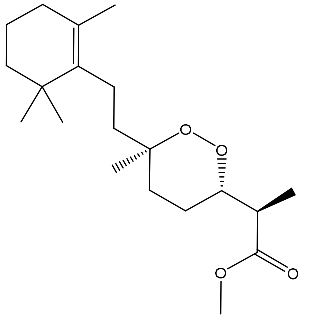	−8.1
124	6′- Hydroxy4′-methoxyavarone **(23)**	*D.cinerea*	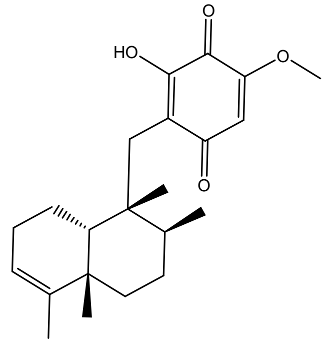	−8.1
125	(22E)-Methylcholesta-5,22-diene-1α,3β,7α-triol **(198)**	*Antipathes dichotoma*	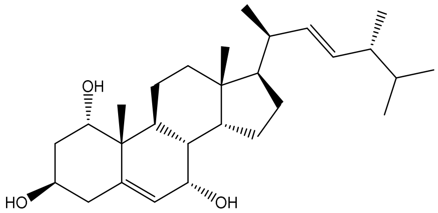	−8.1
126	Neviotine-C **(177)**	*Siphonochalina siphonella*	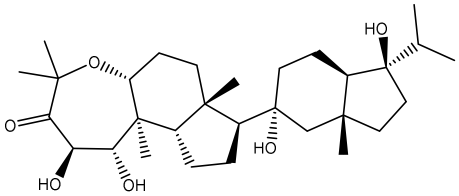	−8.0
127	Sipholenol K **(167)**	*S. siphonella*	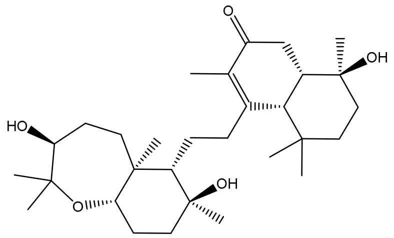	−8.0

128	Trochelioid B **(37)**	*S. trocheliophorum*	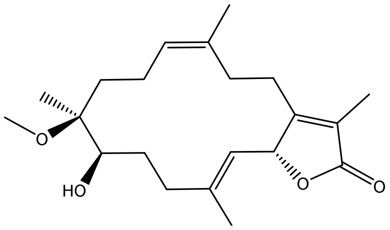	−8.0
129	Xeniolide-E (**76**)	*X. obscuronata*	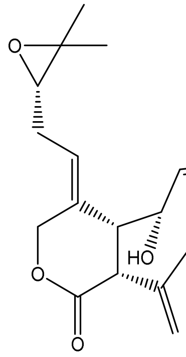	−8.0
130	Epoxyxeniaphyllenol-A **(80)**	*X. lilielae, X. macrosoiculata*	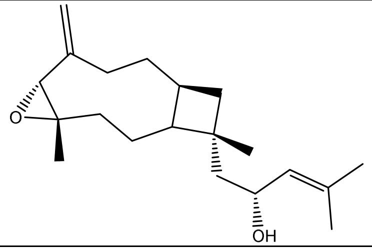	−7.9
131	Xeniaphyllenol-B **(82)**	*X. macrosoiculata*	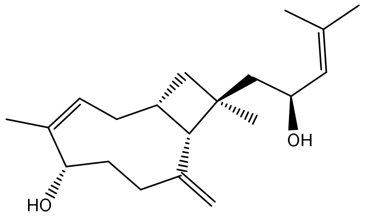	−7.9
132	Chelodane **(107)**	*Chelonaplysilla erecta*	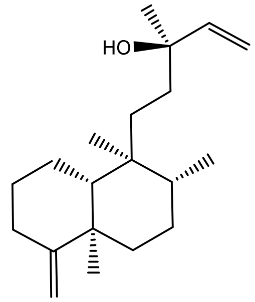	−7.9
133	Aikupikoxide B **(143)**	*D. erythraeanus*	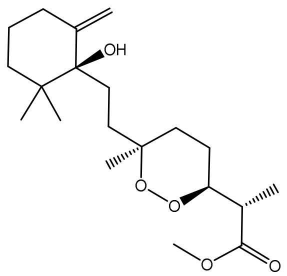	−7.9

134	Eryloside A **(197)**	*Genus Erylus*	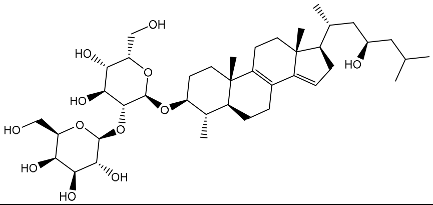	−7.9
135	3-Deoxy-20-acetylpresinularolide B **(64)**	*L. crassum*	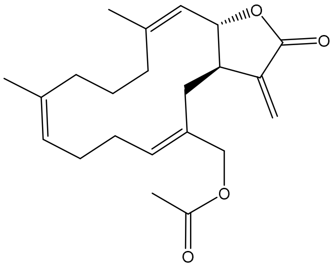	−7.9
136	Sarcophine **(30)**	*S. glaucum*	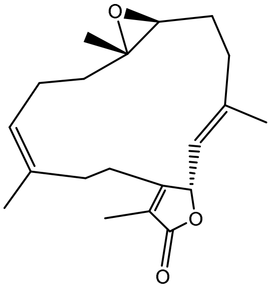	−7.9
137	Xenicin **(73)**	*Xenia macrosoiculata*	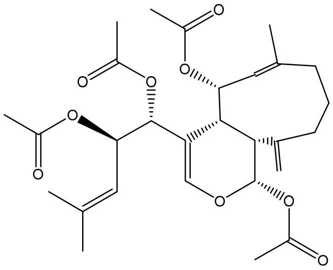	−7.8
138	Sarcophytol M **(65)**	*Litophyton arboreum*	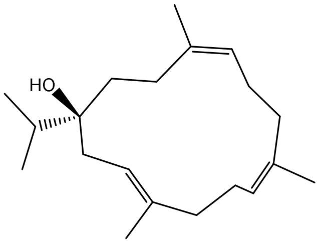	−7.8
139	Smenotronic acid **(26)**	*Smenospongia sp.*	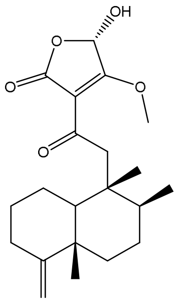	−7.8

140	Zaatirin **(109)**	*C. erecta*	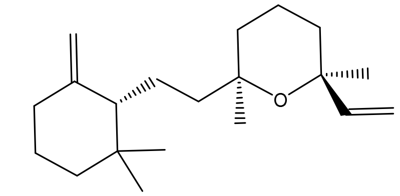	−7.8
141	Cholest-5-en-3β-yl-formate **(204)**	*A. dichotoma*	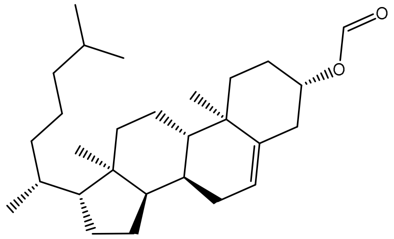	−7.8
142	Pachycladin B **(85)**	*C. pachyclados*	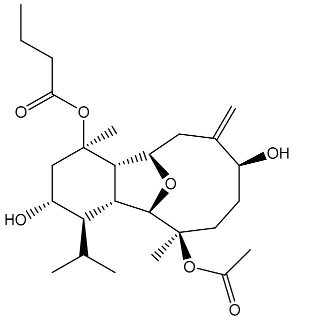	−7.8
143	Sclerophytin A **(93)**	*C. pachyclados*	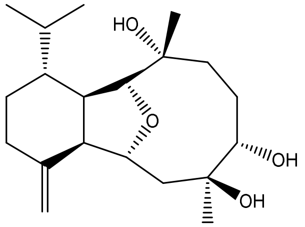	−7.8
144	epi-Sigmosceptrellin B **(147)**	*D. erythraeanus*	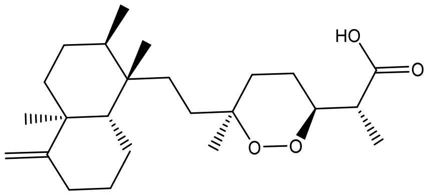	−7.8
145	Pachycladin D **(91)**	*C. pachyclados*	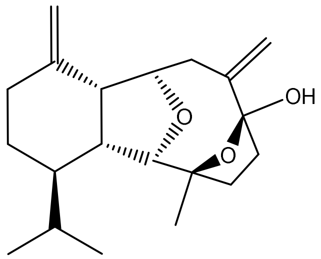	−7.7

146	Barekoxide **(108)**	*C. erecta*	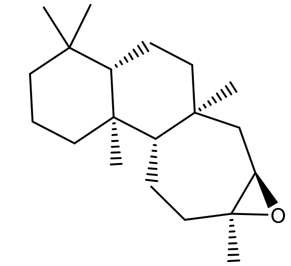	−7.7
147	Sarcophytolide 1 **(32)**	*S. glaucum*	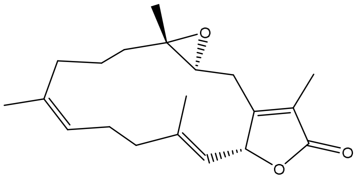	−7.7
148	Biflora-4,10(19),15-triene **(106)**	*X. obscuronata*	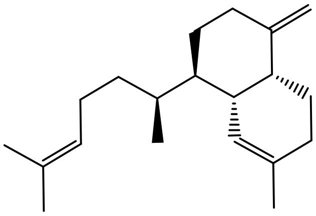	−7.7
149	Aikupikoxide C **(142)**	*D. erythraeanus*	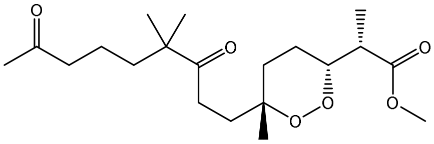	−7.6
150	(+)-7α,8β-Dihydroxydeepoxy-sarcophine **(31)**	*S. glaucum*	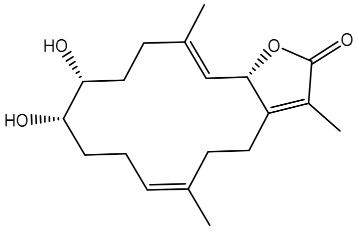	−7.6
151	Scalardysin **(114)**	*Dysidea herbacea*	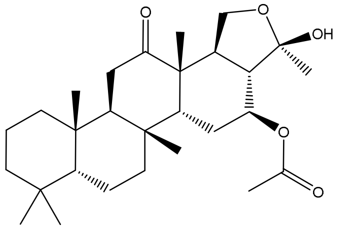	−7.6
152	Avarol **(19)**	*Dysidea cinerea*	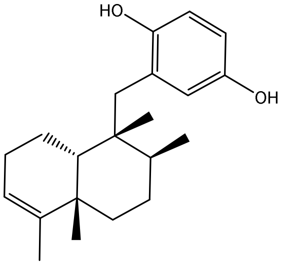	−7.6

153	Sarcophytolide C **(68)**	*S. glaucum*	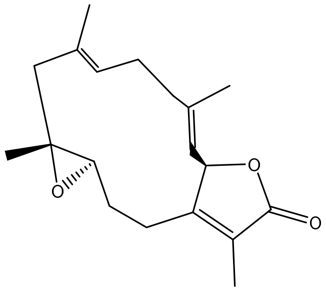	−7.6
154	Sclerophytin F methyl ether **(94)**	*C. pachyclados*	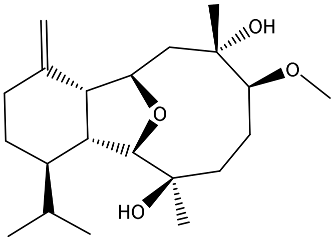	−7.5
155	20-Acetylsinularolide B **(59)**	*L. crassum*	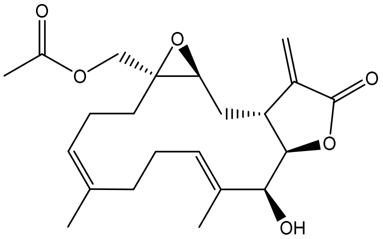	−7.5
156	Pachycladin E **(92)**	*C. pachyclados*	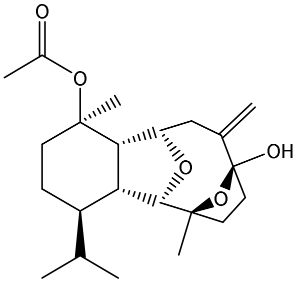	−7.5
157	(1R,2E,4S,6E,8R,11R,12R)-2,6-cembradiene-4,8,11,12-tetrol **(71)**	*S. auritum*	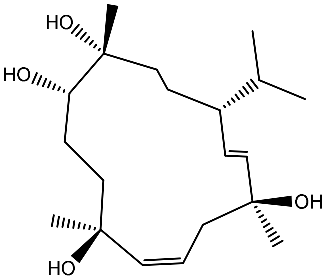	−7.5
158	Xeniaphyllenol-C **(79)**	*X. macrosoiculata*	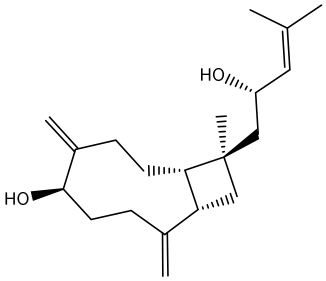	−7.5

159	Cembrene-A **(51)**	*Alcyonium utinomii*	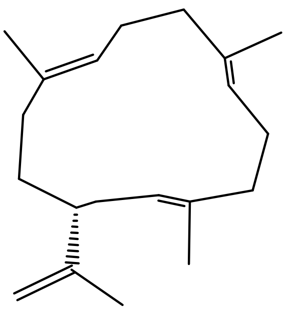	−7.5
160	Triterpene	*Clothraria rubrinoidis*	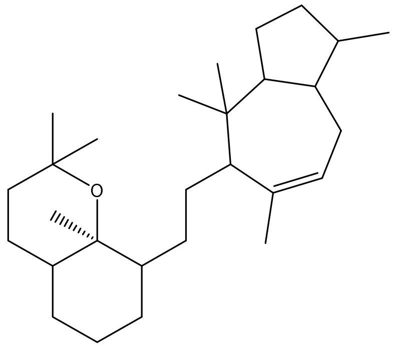	−7.5
161	Sinularolide C **(61)**	*L. crassum*	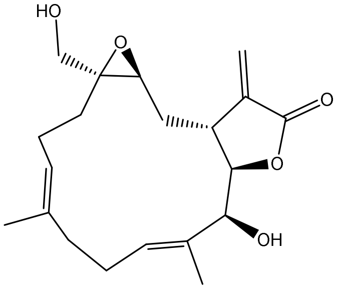	−7.5
162	Obscuronatin **(105)**	*Xenia obscuronata*	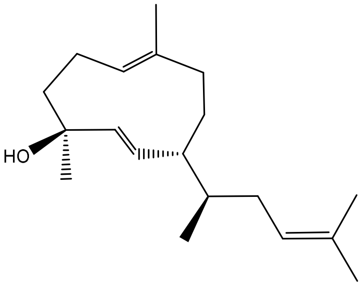	−7.4
163	Cladiellisin **(88)**	*C. pachyclados*	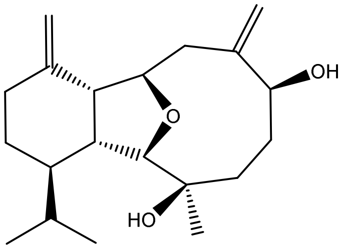	−7.4

164	Nephthenol **(50)**	*Lobophytum pauciflorum*	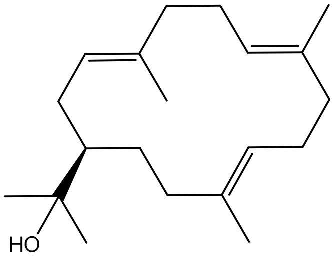	−7.4
165	Seco-norrlandin C **(113)**	*Dysidea sp.*	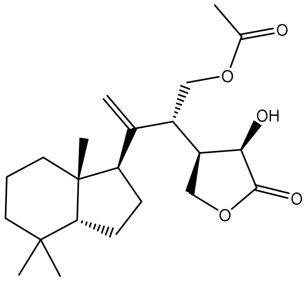	−7.4
166	6′-Hydroxyavarol **(24)**	*D.cinerea*	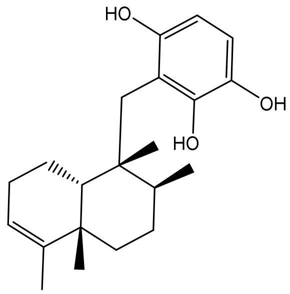	−7.4
167	Alcyonol C **(54)**	*A. utinomii*	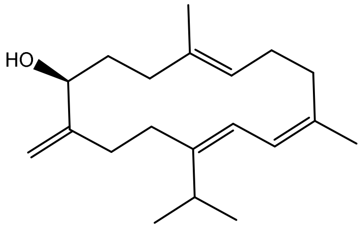	−7.4
168	Pauciflorol B **(56)**	*L. pauciflorum*	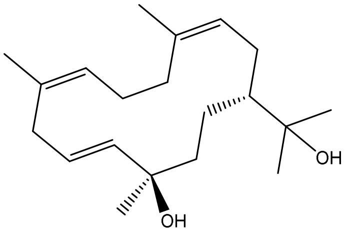	−7.4
169	20-Acetylsinularolide C **(60)**	*L. crassum*	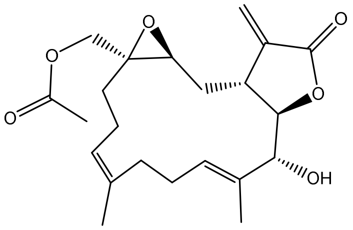	−7.4

170	3′-Hydroxyavarone **(20)**	*D.cinerea*	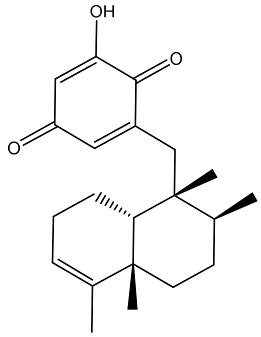	−7.4
171	16-Oxosarcophytonin E **(38)**	*S. trocheliophorum*	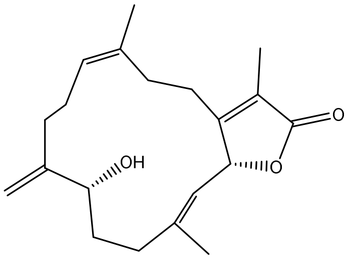	−7.4
172	Alcyonol A **(52)**	*A. utinomii*	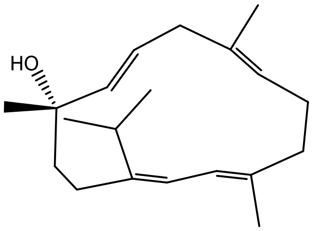	−7.3
173	Gorgostan-3β,5α,6β,11α-tetraol (sarcoaldosterol A) **(180)**	*H. ghardaqensis*	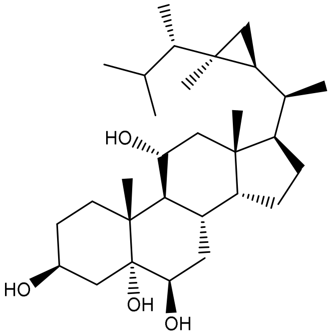	−7.3
174	Norrlandin **(111)**	*Dysidea sp.*	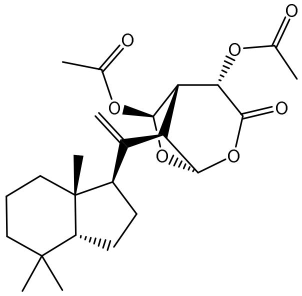	−7.3

175	7β-Acetoxy-8α-hydroxy-deepoxysarcophine **(47)**	*S. glaucum*	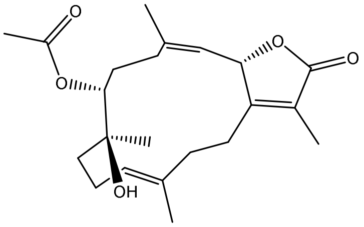	−7.3
176	Sclerophytin B **(95)**	*C. pachyclados*	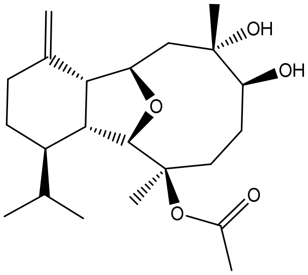	−7.3
177	Ilimaquinone **(18)**	*Smenospongia sp.*	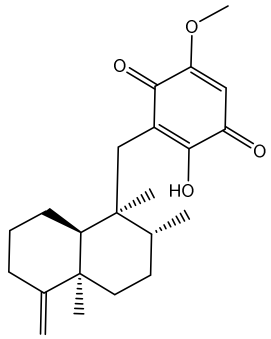	−7.3
178	Alcyonol B **(53)**	*A. utinomii*	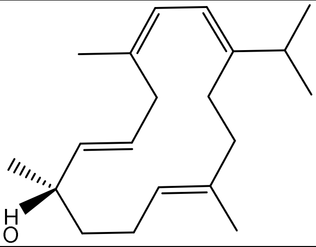	−7.3
179	Sarcophytolide B **(67)**	*S. glaucum*	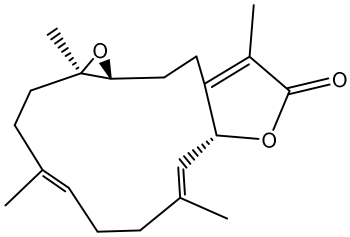	−7.3
180	2-epi-Sarcophine **(70)**	*S. auritum*	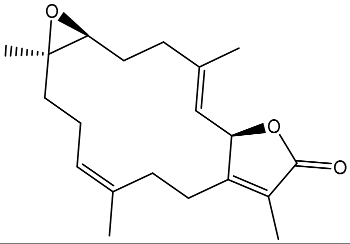	−7.3

181	Aikupikoxide A **(140)**	*D. erythraeanus*	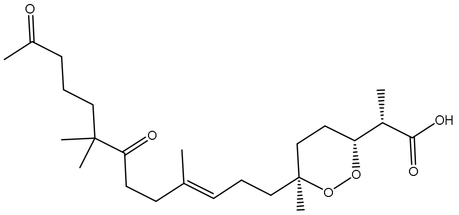	−7.2
182	Pachycladin C **(87)**	*C. pachyclados*	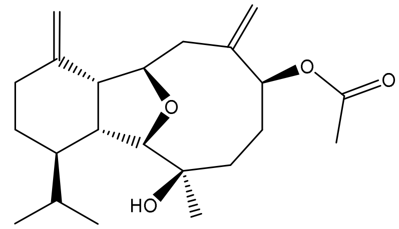	−7.2
183	Ylangene	*Sarcophyton glaucum*	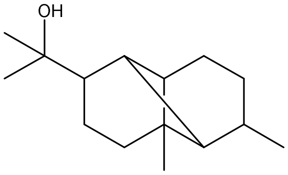	−7.2
184	3-Deoxypresinularolide B **(63)**	*L. crassum*	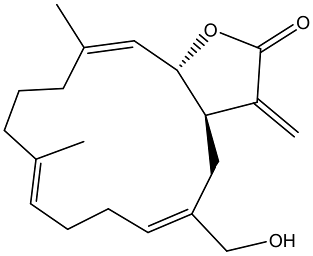	−7.2
185	8-epi-Sarcophinone **(40)**	*S. trocheliophorum*	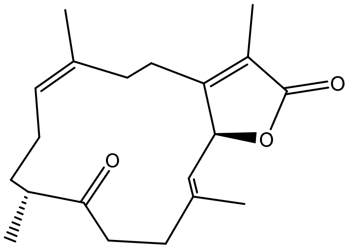	−7.2
186	(1S,2E,4R,7E,11E,13S)-Cembratrien-4,13-diol **(33)**	*S. glaucum*	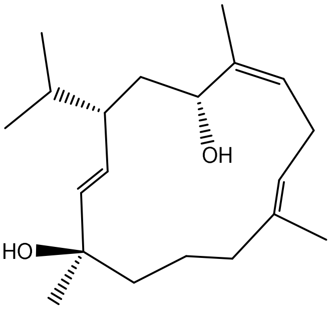	−7.1

187	Alismol **(5)**	*Litophyton arboreum*	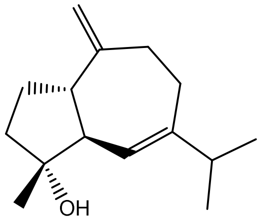	−7.1
188	(2R,7R,8R)-Dihydroxy-deepoxysarcophine **(46)**	*S. glaucum*	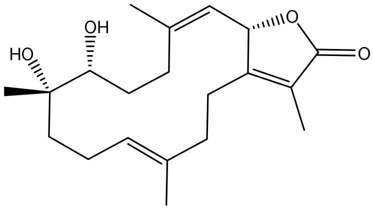	−7.1
189	10-O-Methyl alismoxide **(7)**	*L. arboreum*	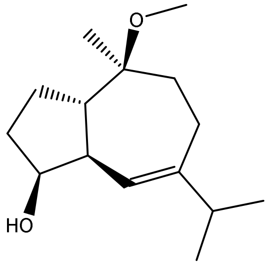	−7.1
190	12(S)-Hydroperoxylsarcoph-10-ene **(45)**	*S. glaucum*	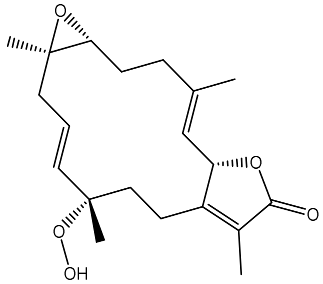	−7.1
191	(1S,2E,4R,6E,8S,11R,12S)-8,11-Epoxy-4,12-epoxy-2,6-cembradiene **(35)**	*S. glaucum*	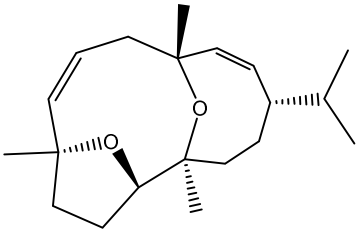	−7.1
192	3-Acetyl Cladiellisin **(89)**	*C. pachyclados*	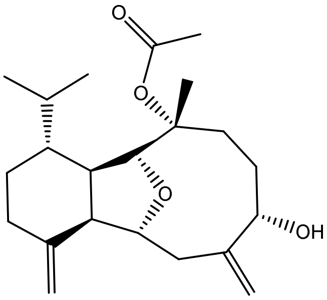	−7.1

193	(1S,2E,4R,6E,8R,11S,12R)-8,12-Epoxy-2,6-cembradiene-4,11-diol **(34)**	*S. glaucum*	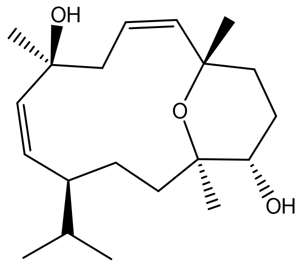	−7.1
194	Cembrene C **(29)**	*Sarcophyton trocheliophorum*	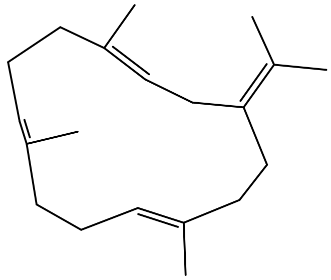	−7.1
195	Singardin (72)	Sinularia gardineri	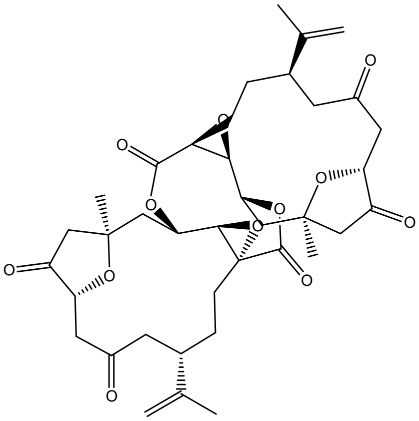	−7.1
196	Aromadendrane **(B)**	*S. regulare*	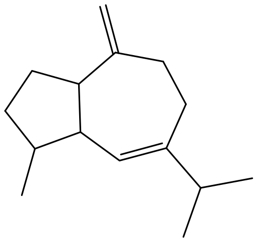	−7.1
197	Xenialactol-D **(74)**	*X. obscuronata*	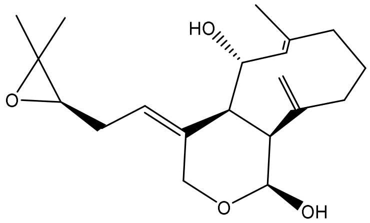	−7.1

198	3′,6′-Dihydroxyavarone **(21)**	*D.cinerea*	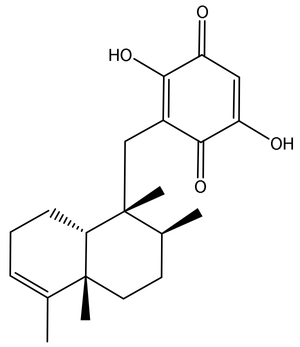	−7.1
199	Deoxosarcophine **(69)**	*S. glaucum*	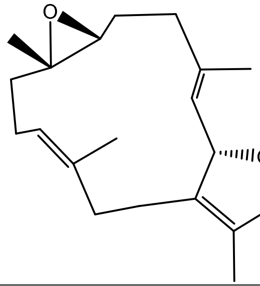	−7.1
200	Dendronephthol A **(1)**	*Nephtheidae*	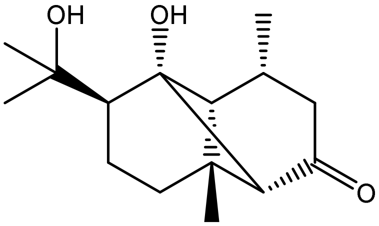	−7.1
201	6′-Acetoxyavarone (22)	D.cinerea	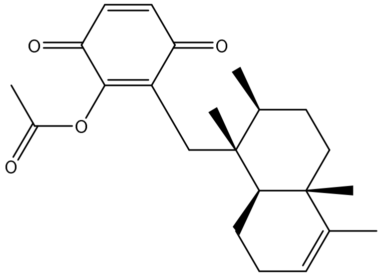	−6.9
202	Hyrtiosenolide A **(13)**	*sponge Hyrtios sp.*	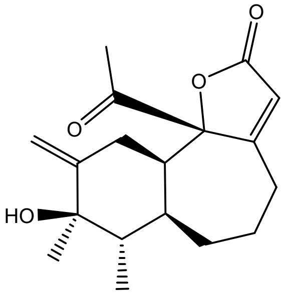	−6.9

203	Cladiella-6Z,11(17)-dien-3-ol **(97)**	*C. pachyclados*	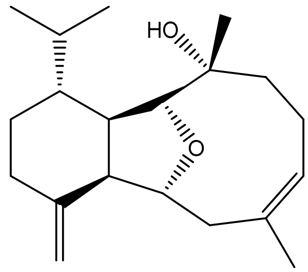	−6.9
204	3,6-Diacetyl Cladiellisin **(90)**	*C. pachyclados*	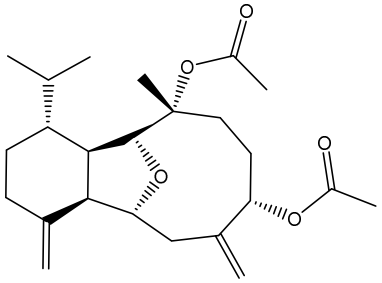	−6.8
205	Guaianediol **(4)**	*Sinularia gardineri*	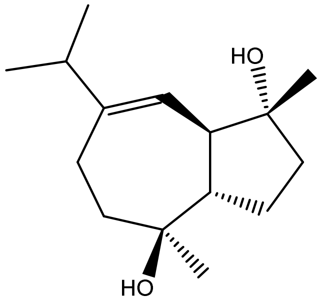	−6.8
206	ent-Sarcophine **(39)**	*S. trocheliophorum*	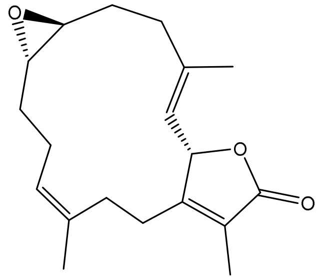	−6.8
207	Durumolide C **(43)**	*Sinularia polydactyla*	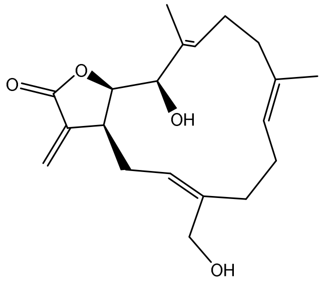	−6.8
208	Pauciflorol A **(55)**	*L. pauciflorum*	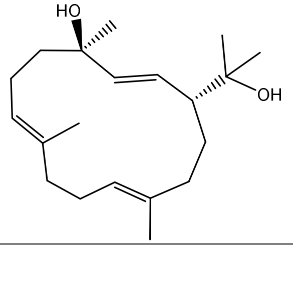	−6.8

209	Alismoxide **(8)**	*L. arboreum*	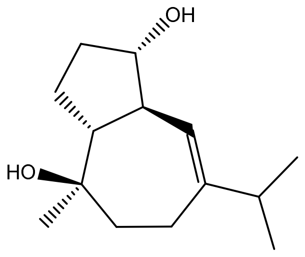	−6.8
210	Xeniaphyllene-dioxide **(78)**	*X. lilielae*	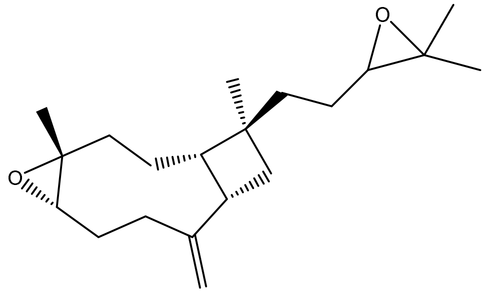	−6.7
211	Juncins B **(100)**	*J. juncea*	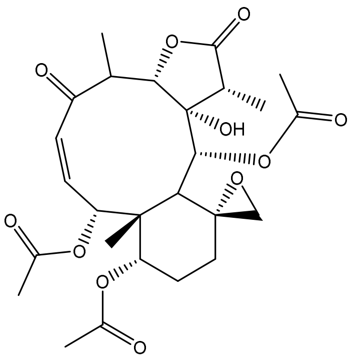	−6.7
212	Palustrol **(9)**	*Sarcophyton trocheliophorum*	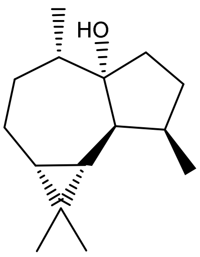	−6. 7
213	Lactiflorenol **(6)**	*Sinularia polydactyla*	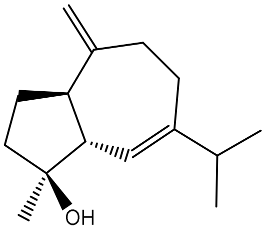	−6. 7

214	Sarcotrocheliol acetate **(41)**	*S. trocheliophorum*	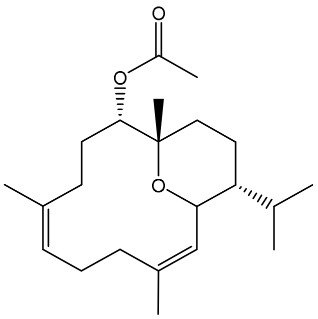	−6.6
215	5-Hydroxy-8-methoxy-calamenene **(15)**	*Parerythropodium fulvum fulvum*	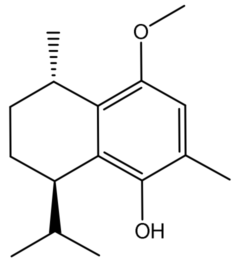	−6.6
216	10(14)-Aromadendrene **(10)**	*Sarcophyton glaucum*	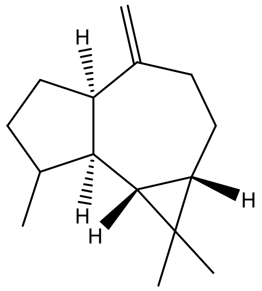	−6.6
217	Dactyltronic acids **(27)**	*Smenospongia sp.*	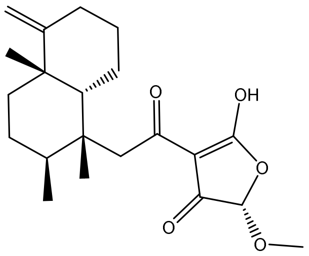	−6.6
218	Dendronephthol C **(3)**	*Nephtheidae*	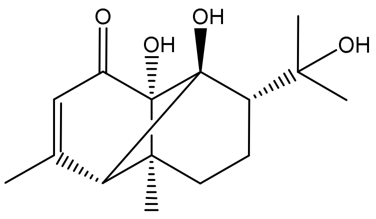	−6.4

219	Juncins **(104)**	*J. juncea*	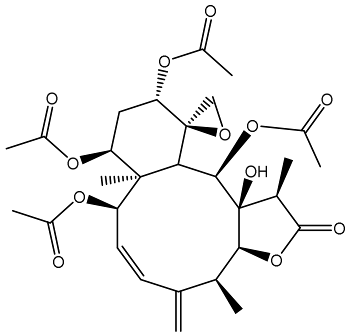	−6.4
220	6′-Acetoxyavarol **(25)**	*D.cinerea*	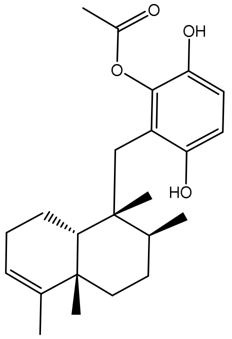	−6.4
221	5-Hydroxy-8-methoxy-calamenene-6-al **(16)**	*Parerythropodium fulvum fulvum*	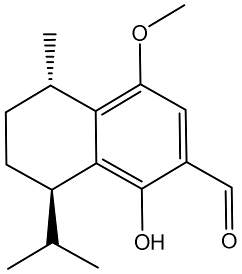	−6.4
222	Erylosides L **(225)**	*E. lendenfeldi*	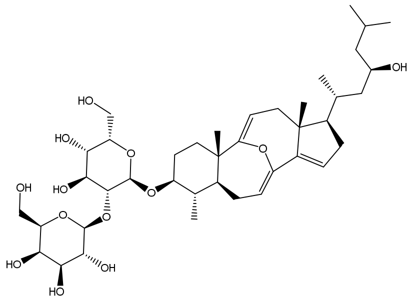	−6.4
223	Sarcophytolol **(66)**	*Sarcophyton glaucum*	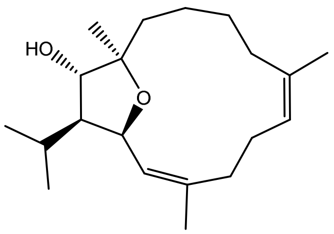	−6.3

224	Fulfulvene **(11)**	*Parerythropodium fulvum fulvum*	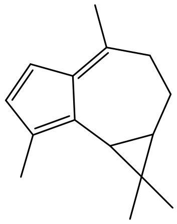	−6.2
225	Dendronephthol B **(2)**	*Nephtheidae*	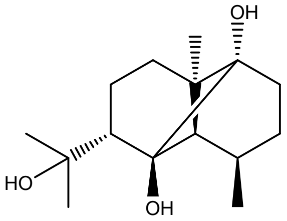	−6.2
226	Aikupikoxide D **(141)**	*D. erythraeanus*	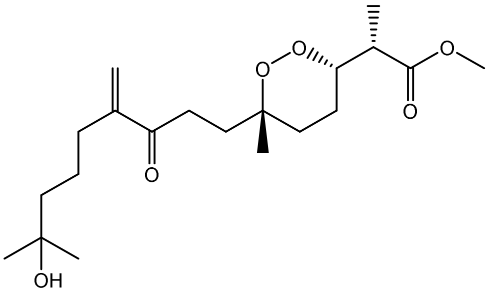	−5.5
227	Briarein A **(98)**	*Junceella juncea*	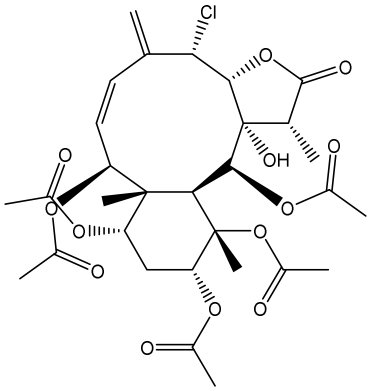	−4.6
228	Juncins C **(101)**	*J. juncea*	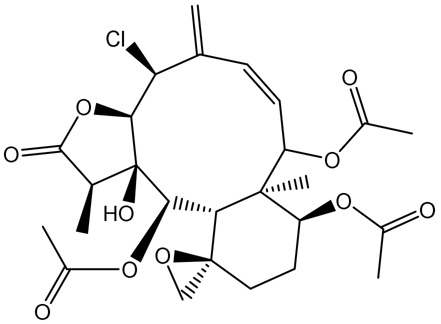	−4.3

Table S2. Computed Autodock and MM/GBSA binding energies (in kcal/mol) for the top 27 potent marine natural products (MNPs) against SARS-CoV-2 main protease (M^{pro}) over 250 ps implicit solvent MD simulations[a].

No.	Compound Name	Autodock Binding Score (kcal/mol)	MM/GBSA Binding Energy (kcal/mol)
1	Lopinavir	−9.8	−39.4
2	Erylosides B **(226)**	−12.1	−50.8
3	Erylosides K **(224)**	−11.1	−50.4
4	3β-Hexadecanoylcholest-5-en-7-one **(202)**	−10.0	−49.3
5	Eryloside A **(197)**	−10.7	−47.8
6	SipholenolA-4-O-3′,4′-dichlorobenzoate **(151)**	−10.5	−42.6
7	Sipholenone E **(163)**	−9.9	−40.9
8	Sipholenone A **(175)**	−11.0	−37.7
9	3β-25-Dihydroxy-4-methyl-5α,8α-epidioxy-2-ketoergost-9-ene **(178)**	−12.2	−36.1
10	Sipholenol D **(176)**	−11.0	−35.9
11	(22R,24E,28E)-5β,6β-Epoxy-22,28-oxido-24-methyl-5αcholestan-3β,25,28-triol **(191)**	−11.4	−35.2
12	Sipholenol I **(174)**	−11.8	−35.1
13	Sipholenol H **(157)**	−12.0	−34.7
14	Sipholenone D **(155)**	−10.7	−33.4
15	Siphonellinol C **(172)**	−11.3	−33.0
16	Neviotine B **(158)**	−10.9	−32.2
17	Tasnemoxide A **(144)**	−11.4	−32.2
18	Siphonellinol-C-23-hydroperoxide **(171)**	−11.2	−31.8
19	Brassicasterol **(222)**	−10.1	−31.4
20	Cholest-5-en-3β,7β-diol **(206)**	−10.3	−31.1
21	Dahabinone A **(162)**	−11.9	−30.2
22	Clionasterol **(219)**	−10.3	−29.9
23	Depresosterol **(190)**	−12.3	−28.1
24	Campesterol **(221)**	−10.3	−27.8
25	Cholesterol **(184)**	−10.3	−27.4
26	Lobophytosterol **(188)**	−11.5	−27.4
27	24-Methylcholestane-5-en-3β,25-diol **(187)**	−10.6	−27.3
28	Stigmasterol **(220)**	−10.5	−26.4

[a]Data sorted according to the calculated MM/GBSA binding energies.

Table S3. Network topological analysis for the predicted targets for erylosides B (**226**).

Name	BetweennessCentrality	ClosenessCentrality	Degree	Number Of Undirected Edges
**VEGFA**	0.16167593622379026	0.543859649122807	30	30
**DRD2**	0.09010641139487129	0.4946808510638298	27	27
**STAT3**	0.08946266709776997	0.5166666666666666	24	24
**JUN**	0.0981884572759623	0.510989010989011	23	23
**ADRA2A**	0.024166127699934013	0.45365853658536587	23	23
**ADRA2C**	0.017619738652461147	0.44285714285714284	22	22
**F2**	0.06340725344246699	0.4696969696969697	21	21
**ADRA2B**	0.016564892030468053	0.4407582938388625	21	21
**SLC6A3**	0.02851326445210035	0.44497607655502397	20	20
**ADRA1A**	0.008093604972074064	0.41150442477876104	19	19
**ADRA1B**	0.013961744615366147	0.4246575342465753	19	19
**REN**	0.060652471264093046	0.484375	19	19
**PRKCA**	0.06205750483643582	0.48186528497409326	18	18
**DRD3**	0.008419520597214505	0.43457943925233644	18	18
**OPRK1**	0.019575984856832866	0.42081447963800905	18	18
**ADRA1D**	0.005035012045256654	0.40611353711790393	18	18
**EDNRB**	0.040084345951539736	0.44497607655502397	18	18
**IL2**	0.0567559135447914	0.47692307692307695	17	17
**HTR2A**	0.006128365201270778	0.4096916299559471	17	17
**FGF2**	0.023107182275248352	0.484375	16	16
**PRKCB**	0.030044228440275655	0.4696969696969697	16	16
**HTR2C**	0.004745649753776972	0.40086206896551724	16	16
**SLC6A2**	0.012665535612403297	0.39914163090128757	16	16
**ACHE**	0.017816808762018056	0.41333333333333333	16	16

Table S4. Top 20 most relevant pathways for erylosides B (**226**) targets resulted from Pathway Enrichment Analysis (PEA). PEA was performed using a binomial test and p-values were False Discovery Rate (FDR)-corrected for multiple testing.

Pathway name	Entities found	Entities total	Interactors found	Interactors total	Entities ratio	Entities pValue	Entities FDR	Reactions found	Reactions total	Reactions ratio
Signaling by GPCR	15	1497	9	1666	0.1016	0.00157934	0.0728	45	445	0.03363822
GPCR downstream signalling	14	1355	3	1157	0.092	5.72E-04	0.0353	31	260	0.01965379
GPCR ligand binding	13	665	6	581	0.0451	1.10E-06	1.48E-04	14	185	0.01398443
Class A/1 (Rhodopsin-like receptors)	13	475	6	552	0.03226	1.21E-07	2.69E-05	14	158	0.01194346
Amine ligand-binding receptors	10	88	4	50	0.0059	3.39E-13	2.27E-10	6	22	0.00166301
G alpha (q) signalling events	8	283	3	461	0.0192	1.70E-04	0.0161	7	35	0.0026457
Platelet activation, signaling and aggregation	8	296	2	669	0.0201	7.93E-04	0.0436	15	115	0.00869302
Adrenoceptors	6	48	1	35	0.0032	2.97E-08	9.94E-06	3	7	5.29E-04
G alpha (z) signalling events	5	62	0	48	0.0042	4.02E-06	4.46E-04	6	13	9.83E-04
Interleukin-4 and Interleukin-13 signaling	5	216	1	153	0.0146	0.00110401	0.0563	21	47	0.0035528
POU5F1 (OCT4), SOX2, NANOG activate genes related to proliferation	4	21	2	132	0.0014	3.29E-04	0.0273	4	16	0.00120946
Response to elevated platelet cytosolic Ca2+	4	144	0	104	0.0097	0.00206465	0.0802	4	14	0.00105828
Transcriptional regulation of pluripotent stem cells	4	45	2	231	0.003	0.00300441	0.1051	6	35	0.0026457

Platelet Aggregation (Plug Formation)	4	53	1	315	0.0036	0.00778789	0.1999	4	27	0.00204097
Adrenaline signalling through Alpha-2 adrenergic receptor	3	5	0	0	3.40E-04	2.05E-07	3.42E-05	1	1	7.56E-05
G alpha (12/13) signalling events	3	87	0	63	0.0059	0.00402801	0.1329	3	15	0.00113387
VEGFR2 mediated cell proliferation	3	31	0	132	0.0021	0.00556703	0.1725	7	12	9.07E-04
Dopamine receptors	2	6	3	9	4.08E-04	4.53E-04	0.0335	2	3	2.27E-04
Depolymerisation of the Nuclear Lamina	2	23	0	8	0.0015	0.00216767	0.0802	2	6	4.54E-04
RUNX1 and FOXP3 control the development of regulatory T lymphocytes	2	17	2	39	0.0011	0.00637006	0.1771	6	20	0.00151183
